# Osmotically Tunable Microdroplets Enable Amplification‐Free CRISPR Detection of Gene Doping

**DOI:** 10.1002/advs.202515861

**Published:** 2025-10-14

**Authors:** Jihun Han, Reya Ganguly, Joon‐Yeop Yi, Hyewon Yun, So‐Yeon Jung, Changmin Sung, Chang‐Soo Lee

**Affiliations:** ^1^ Department of Chemical Engineering and Applied Chemistry Chungnam National University Yuseong‐gu Daejeon 34134 Republic of Korea; ^2^ Interdisciplinary Program of Bioengineering Seoul National University Seoul 08826 Republic of Korea; ^3^ Doping Control Center Korea Institute of Science and Technology Seoul 02792 Republic of Korea

**Keywords:** amplification‐free detections, double emulsions, gene doping, microfluidics, osmosis

## Abstract

Gene doping is an increasing challenge in sports, demanding highly sensitive and specific detection tools beyond the limitations of the current amplification‐dependent methods. Here, an innovative amplification‐free clustered regularly interspaced short palindromic repeats/CRISPR‐associated protein (CRISPR/Cas) 12a assay integrated with osmotically tunable double emulsion (DE) droplets is reported for rapid and ultrasensitive gene doping detection. Target DNA and CRISPR/Cas12a complexes are encapsulated within DE droplets, where osmotic shrinkage rapidly concentrates the reaction components, thereby enhancing the fluorescent signal intensity without nucleic acid amplification. This platform enables the detection of the human erythropoietin (*hEPO*) gene at unprecedented attomolar levels within 30 min, achieving a 25‐fold improvement in sensitivity compared with that of nonshrinkable formats. Notably, the assay demonstrated a robust and specific performance in complex serum samples with minimal matrix interference. This novel approach offers a rapid, reliable, and inherently contamination‐free solution for gene doping surveillance with broad potential for versatile amplification‐free nucleic acid diagnostics.

## Graphical abstract

A novel amplification‐free clustered regularly interspaced short palindromic repeats/CRISPR‐associated protein (CRISPR/Cas) 12a assay is integrated with osmotically shrinkable double emulsion droplets for the rapid and ultrasensitive detection of gene doping. Controlled droplet shrinkage concentrates cleaved reporters, significantly enhancing signal intensity and achieving a 25‐fold improved detection limit, independent of nucleic acid amplification.

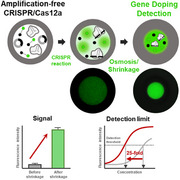

## 1 Introduction

Gene doping refers to the misuse of gene therapy to alter gene expression using polymers or oligomers of nucleic acids, nucleic acid analogs, or genome sequence modifications.^[^ ^1^, ^2^ ^]^ These manipulations can be achieved by administering exogenous genes, transgenes, antisense oligonucleotides, and small‐interfering RNAs, or using genome editing tools, such as clustered regularly interspaced short palindromic repeats (CRISPR) to modify inherited genomes in germline cells.^[^ ^3^, ^4^, ^5^, ^6^ ^]^ Several human genes, including follistatin (*FST*), growth hormone 1 (*GH1*), growth hormone‐releasing hormone (*GHRH*), insulin‐like growth factor 1 (*IGF1*), and erythropoietin (*EPO*), are promising candidates for gene doping.^[^ ^7^ ^]^ These genes encode proteins that enhance muscle size, strength, and endurance, and provide a competitive advantage in sports. Athletes may resort to gene doping with these genes as an alternative to traditional drug doping, particularly given the widespread detection of anabolic substances in conventional anti‐doping tests.^[^ ^8^, ^9^, ^10^, ^11^ ^]^ However, proteins expressed through gene doping are identical to endogenous proteins, potentially allowing athletes to bypass anti‐doping tests that often rely on identifying the recombinant features of protein‐based drugs. Although substantial challenges and ethical concerns persist, the potential misuse of gene technology continues to threaten sports integrity. In response to this emerging risk, the World Anti‐Doping Agency (WADA) included gene and cell doping in its Prohibited List in 2003.^[^ ^12^ ^]^ The current definition of gene doping includes the use of technologies that enhance athletic performance by adding genes or genetic elements, editing genes, or adding cells.^[^ ^13^ ^]^ Gene doping detection has evolved with the development of advanced molecular techniques. Polymerase chain reaction (PCR)‐based methods are regarded as the gold standard, owing to their high accuracy and reproducibility.^[^ ^14^ ^]^ These methods specifically identify unique exon‐exon junctions present in transgenes, distinguishing them from endogenous genes.^[^ ^15^ ^]^ Quantitative real‐time PCR (qPCR) and digital PCR (dPCR) are widely used, with specific primers and probes tailored for transgene detection.^[^ ^11^, ^16^, ^17^, ^18^, ^19^ ^]^ However, despite their high sensitivity and specificity, these methods are often limited by complex protocols, the risk of cross‐contamination, and the requirement for skilled operators. Another promising approach is sequencing‐based methods, such as next‐generation sequencing (NGS), which allows the direct identification of transgenes with high resolution.^[^ ^20^, ^21^ ^]^ Despite its high potential, NGS requires expensive equipment and skilled operators, which limits its current use for on‐site detection.^[^ ^22^, ^23^ ^]^ Additionally, the CRISPR/Cas system has recently emerged as an innovative solution for gene doping detection.^[^ ^24^, ^25^, ^26^, ^27^, ^28^, ^29^ ^]^ These systems use the specificity of CRISPR‐associated nucleases to target and detect genetic sequences.^[^ ^30^, ^31^ ^]^ However, these systems traditionally use fluorescence or colorimetric analysis tools with lower sensitivity, requiring typical involvement of amplifying the target nucleic acid using techniques, such as PCR, loop‐mediated isothermal amplification (LAMP), and recombinase polymerase amplification (RPA), followed by identifying the amplicons using guide crRNA.^[^ ^32^, ^33^ ^]^ These approaches involve complex assay mixtures, specialized experimental equipment, primer optimization, and reverse transcriptase, making them prone to false‐positive results and unsuitable for on‐site DNA diagnostics.

To overcome these limitations, several amplification‐free CRISPR/Cas‐based detection strategies have been developed.^[^ ^34^, ^35^, ^36^ ^]^ One of the fundamental approaches is to optimize crRNAs and reporters, which are critical for detection performance.^[^ ^37^, ^38^, ^39^, ^40^, ^41^, ^42^, ^43^ ^]^ For example, increasing the number of recognition sites improves amplification‐free CRISPR/Cas detection signals such as engineered DNA barcode complexes with multiple recognition sites for the Cas12a/crRNA complex were designed for an ELISA‐like immune‐CRISPR assay.^[^ ^37^ ^]^ Additionally, signal reporters, such as hairpin DNA structures, have been designed to improve trans‐cleavage efficiency and amplify fluorescence signals.^[^ ^39^ ^]^ Nanomaterial‐based reporters, including gold nanoparticles, further enhance detection sensitivity by providing fluorescence quenching and signal amplification capabilities.^[^ ^44^, ^45^, ^46^ ^]^ A second approach uses droplets to compartmentalize the reaction as a digital CRISPR detection platform.^[^ ^47^, ^48^ ^]^ This method isolates the CRISPR reaction into discrete units, significantly increasing the localized concentration of target molecules. In digital assays, a sample is divided into multiple individual partitions, each of which has a different number of biological targets (e.g., 0, 1, 2, or 3). Each partition is an independent microreactor, and the partition containing the target produces an increased fluorescent signal, allowing absolute quantification using a Poisson distribution. This results in improved sensitivity compared with that of bulk CRISPR assays, which is attributed to the localized concentration increase of the target molecules achieved through partitioning.^[^ ^49^ ^]^ Another strategy involves the development of a signal transducer in which traditional fluorescence‐based readouts are replaced with electrochemical,^[^ ^50^ ^]^ surface‐enhanced Raman spectroscopy (SERS), or nanopore‐based sensing systems.^[^ ^51^, ^52^ ^]^ Electrochemical sensors measure current changes caused by CRISPR activity, while SERS‐based platforms use metal nanostructures to amplify Raman signals, achieving ultrasensitive detection. Similarly, nanopore biosensors rely on resistance changes induced by CRISPR/Cas‐mediated interactions with target molecules, thereby offering highly specific detection capabilities. Finally, the cascade signal amplification system aims to amplify the output signals generated by CRISPR/Cas activity without pre‐amplifying the target nucleic acid.^[^ ^53^, ^54^, ^55^ ^]^ These systems use enzymatic cascades or nucleic acid circuits to enhance signal output, thereby improving sensitivity.^[^ ^56^, ^57^ ^]^ For instance, the incorporation of DNA barcodes or multiple recognition sites for Cas enzymes enables the generation of amplified fluorescence or electrochemical signals, further extending the detection limits.

Although these amplification‐free strategies demonstrate the potential of CRISPR‐based diagnostics, they still face substantial challenges in real‐world applications. For example, nanopore sensors provide a direct readout of CRISPR‐mediated cleavage events with single‐molecule sensitivity, but rely on costly instrumentation and complex signal interpretation. Electrochemical platforms offer cost‐effective and portable solutions; however, their performance is frequently constrained by electrode surface modifications and susceptibility to background noise in biological samples. SERS‐based systems can achieve ultrasensitive performance; however, their dependence on sophisticated nanostructures and specialized equipment limits their widespread applicability. Similarly, conventional digital droplet methods enhance sensitivity by partitioning the sample; however, they do not actively amplify the fluorescent signal within each positive microreactor, which can remain weak for low‐abundance targets. Collectively, these approaches highlight the difficulty in achieving an optimal balance between sensitivity, speed, cost, and operational simplicity.

To address the fundamental limitations of sensitivity and signal‐to‐noise ratio (SNR), we used double emulsion (DE) droplets as a dynamic micro‐platform capable of enhancing detection signals. DE droplets, typically generated as water‐in‐oil‐in‐water (W/O/W) structures through microfluidic‐based sequential emulsification, possess a unique core‐shell architecture that compartmentalizes and isolates the reaction volumes.^[^ ^58^, ^59^, ^60^ ^]^ Their versatility as microreactors has been extensively demonstrated in diverse applications, including high‐throughput screening, single‐cell analysis, and controlled drug delivery.^[^ ^61^, ^62^, ^63^ ^]^ However, conventional DE systems still face challenges in terms of long‐term stability and have primarily been used as passive containers. The opportunity to leverage the core‐shell structure as a functional semipermeable membrane, capable of dynamically concentrating internal contents and directly amplifying detection signals without additional biochemical reactions, remains relatively underexplored, yet represents a promising strategy to overcome the sensitivity barriers of amplification‐free diagnostics.

Here, we present a novel approach that integrates the CRISPR/Cas12a technique with W/O/W DE droplets to develop an amplification‐free assay platform for detecting the human EPO (*hEPO)* gene in gene doping tests. The shell of the DE droplet functions as a semipermeable membrane, allowing selective water permeation while preventing the diffusion of high‐molecular‐weight substances (e.g., fluorescent reporters). This property enables precise tuning of the core volume of the DE droplets via osmosis. When the osmotic pressure of the outer phase exceeds that of the inner phase, the solvent exits the inner aqueous core, causing shrinkage of the DE droplets, and vice versa. Droplet shrinkage leads to an increased concentration of fluorescent molecules in the core, thereby enhancing signal intensity.

This approach enabled the generation of shrinkable DE droplets encapsulating CRISPR/Cas12a, creating a highly sensitive and signal‐enhancing platform regulated through osmosis. Our method facilitates the rapid CRISPR‐mediated detection of gene doping and achieves robust single‐molecule quantification without the need for target pre‐amplification. We further validated the feasibility of detecting gene doping by demonstrating the high sensitivity and specificity of our method by spiking of the *hEPO* gene into serum samples.

This unique strategy has several advantages. First, it is a purely physical signal enhancement mechanism that avoids the need for complex electronic readouts, significantly reduces cost, and simplifies the workflow. Second, by rapidly concentrating the fluorescent products after the enzymatic reaction, it directly boosts the SNR in a manner that conventional droplet methods cannot. Finally, the entire process occurs within the stable, enclosed environment of a DE droplet, inherently protecting the assay from matrix effects that can plague surface‐based sensors. This integration provides a uniquely robust, accessible, and powerful solution for ultra‐sensitive diagnosis.

## 2 Results

### 2.1 Overview of Amplification‐Free Rapid and Ultra‐Sensitive Detection of Gene Doping

**Figure 1:**
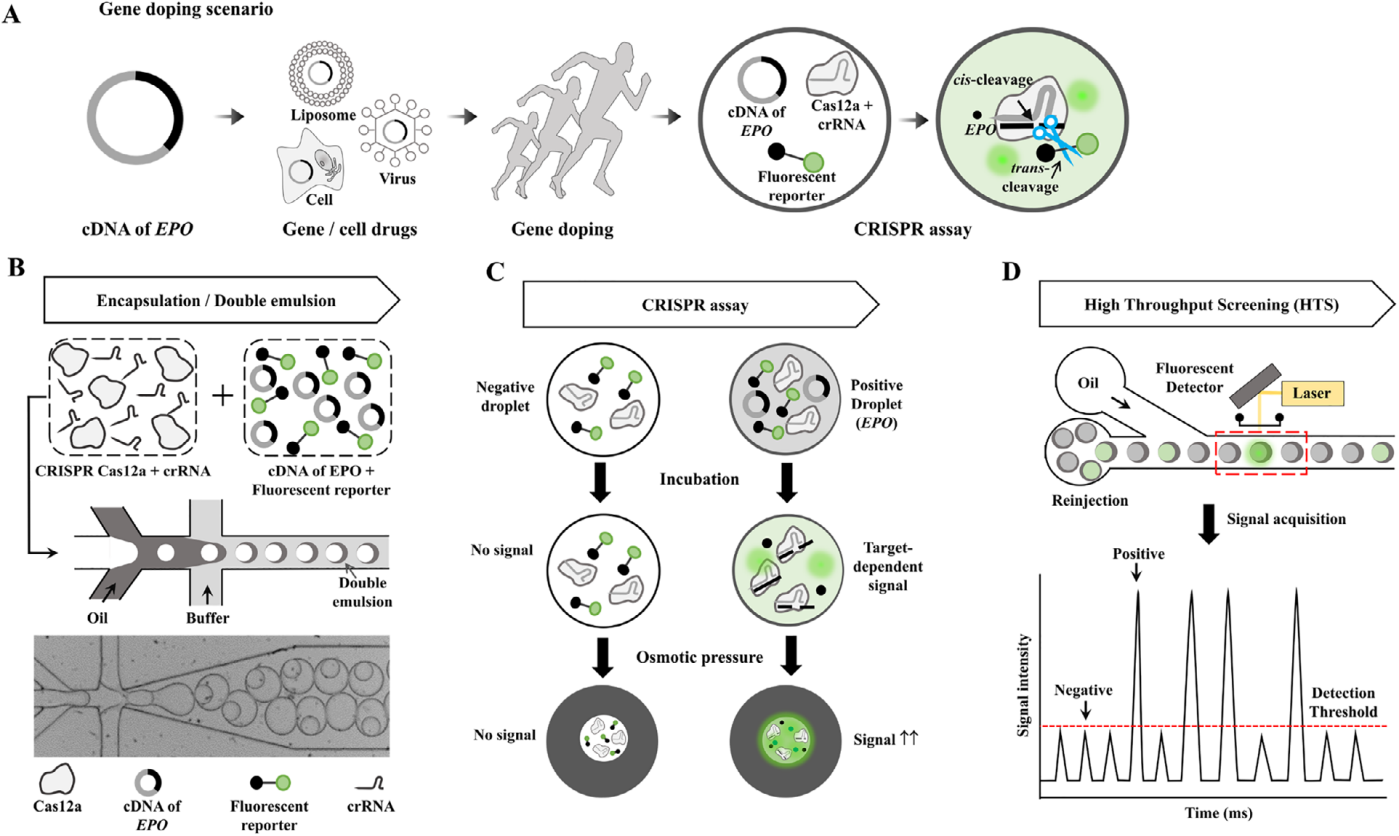
Workflow for amplification‐free detection of gene doping using shrinkable DE droplets. A) Overview of gene doping and the detection process. A schematic representation of the sequential steps involved in gene doping detection. The cDNA of *hEPO* is used to illegally enhance athletic performance. Cells, liposomes, or viruses serve as vectors to deliver *hEPO* cDNA into the host. Blood samples collected from athletes with elevated *hEPO* transfection levels are encapsulated in DE droplets, where the CRISPR‐Cas12a‐based platform generates target‐dependent fluorescent signals. B) The generation of DEs and encapsulation. DE droplets are generated in a microfluidic device. Each droplet encapsulates a reaction mixture comprising the *hEPO* target gene, CRISPR‐Cas12a protein, crRNA, and a fluorescent reporter. These droplets serve as isolated reaction chambers for target detection. C) CRISPR assay and signal enhancement. During incubation, each droplet functions as an independent microreactor for the CRISPR‐Cas12a reaction, producing fluorescence signals upon the detection of *hEPO*, the gene. Additionally, osmotic shrinkage of the DEs increases the signal by concentrating the fluorescence reporters within the droplets. D) Signal detection and sorting. For high‐throughput detection, the DE droplets are reinjected into a microfluidic sorting device. As droplets pass through the microfluidic channel, they are illuminated by a laser, generating measurable fluorescence signals. The schematic shows the fluorescence intensity distribution of positive and negative droplets before and after shrinkage. DE, double emulsion; EPO, erythropoietin.

An ultrasensitive amplification‐free gene doping platform was developed by integrating a CRISPR/Cas12a assay with a shrinkable DE system (**Figure** **1**). Figure 1A illustrates the overall concept of gene doping, using EPO cDNA as an example. Artificial exogenous *hEPO* can be delivered to the body via liposomes, viruses, or engineered cells to enhance EPO expression. This approach increases hemoglobin and hematocrit levels, ultimately improving oxygen transport in the tissue and enhancing athletic performance.

To detect this gene doping event, we used a CRISPR/Cas12a‐based assay. The Cas12a enzyme and its associated crRNA specifically recognized the *hEPO* target (Figure 1A). Once the target was bound, Cas12a underwent cis‐cleavage of DNA and triggered trans‐cleavage of the fluorescent reporter, thereby producing a detectable signal. This mechanism allowed the rapid detection of gene doping without the need for nucleic acid amplification. As schematically illustrated in Figure 1, we integrated droplet microfluidics with CRISPR/Cas12a techniques to detect the target DNA using an amplification‐free approach. This method offers a powerful solution for nucleic acid detection and quantification, owing to its high specificity, sensitivity, and simplicity. However, traditional water‐in‐oil (W/O) single‐emulsion droplets often have stability issues that compromise assay accuracy and reproducibility. In contrast, W/O/W DE droplets offer enhanced stability and reduced leakage, owing to their core‐shell structure, which provides a robust platform.^[^ ^64^, ^65^, ^66^, ^67^, ^68^ ^]^ To implement the droplet format in this study, a flow‐focusing microfluidic device with spatially patterned wettability ^[^ ^69^ ^]^ was used to produce DE droplets (Figure S1, Supporting Information). Our system generated ≈5000 monodisperse single‐core DE droplets per minute. The inner aqueous phase of each DE consisted of two co‐injected streams: one containing CRISPR‐Cas12a and crRNA, and the other containing cDNA and an EPO‐specific fluorescent reporter (Figure 1B).

Each DE functioned as an individual microreactor, in which the CRISPR/Cas12a reaction was carried out at 37 °C for 5 min. These droplets were used to discriminate between the presence and absence of target DNA. For example, in droplets lacking the target DNA (negative droplets), CRISPR/Cas12a remained inactive, resulting in no detectable fluorescence. In contrast, in positive droplets containing the target, the CRISPR/Cas12a complex recognized and cleaved DNA, activating the fluorescent reporter and generating a clear fluorescence signal that distinguished it from the negative droplets. Furthermore, we applied osmotic pressure to induce droplet shrinkage, thereby concentrating fluorescent molecules within the droplet core and enhancing the signal intensity. Positive droplets exhibited significantly higher fluorescence than that of negative droplets, enabling the rapid and sensitive detection of gene doping events (Figure 1C). After the reaction, the DE droplets were reinjected into a microfluidic sorter equipped with a laser‐induced fluorescence detection system. As each droplet passed through the detection zone, the laser excited any fluorescent molecules present, and the resulting emissions were measured in real time. Droplets containing the target gene generated signals above a defined threshold (positive), whereas those lacking the target remained below the threshold (negative). By recording the fluorescence intensity over time, this setup enabled rapid and high‐throughput discrimination between positive and negative droplets, sequentially analyzing up to 18 000 DEs per second (Figure 1D; Figure S1, Supporting Information). Overall, the entire workflow from DE generation to detection for gene doping analysis required only 45 min, with the core CRISPR assay itself requiring only 5 min.

### 2.2 Microfluidic Generation of Shrinkable DE Droplets and Their Characterization

**Figure 2:**
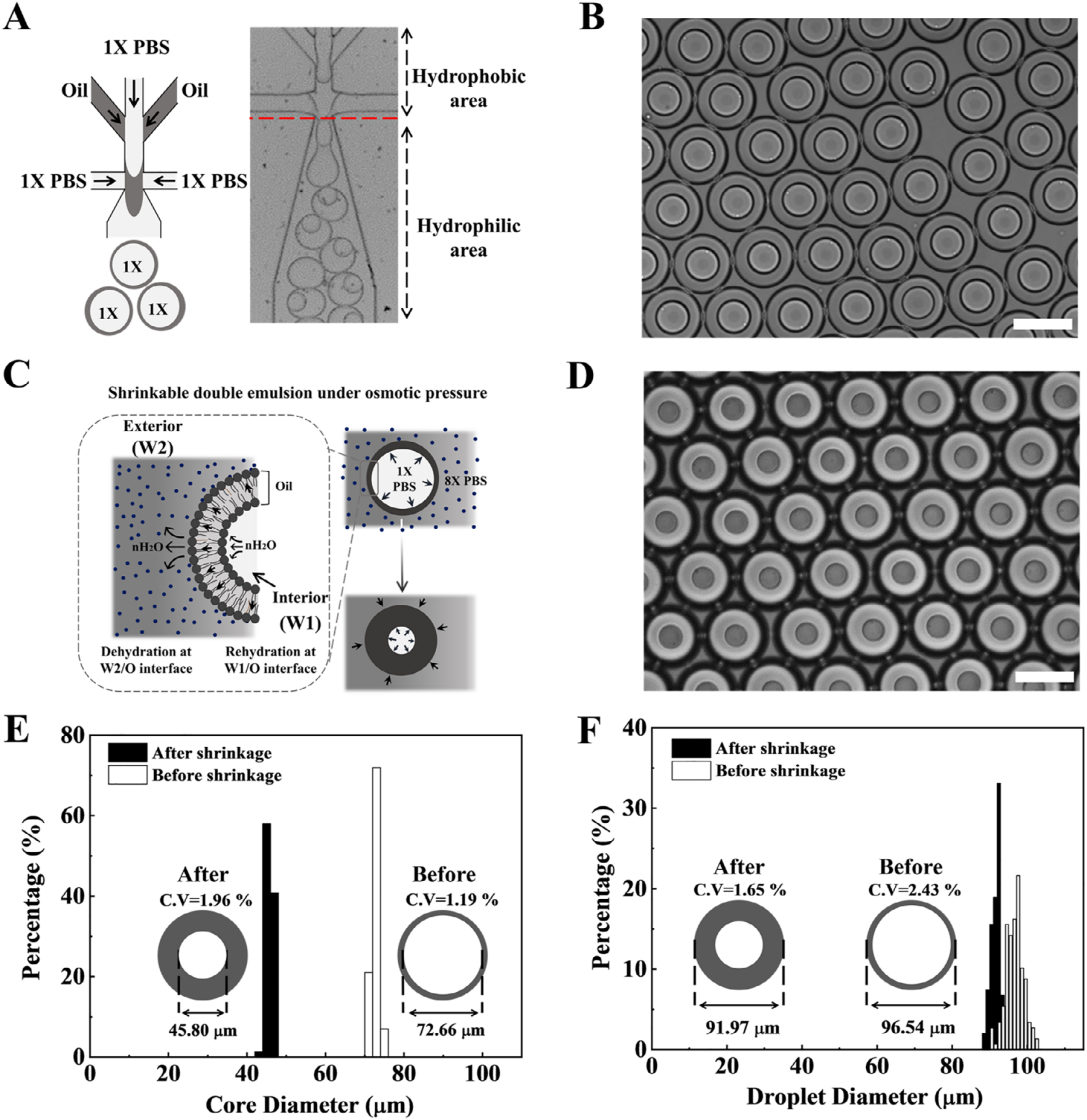
The principle of the generation and the shrinkage of the DE (W1/O/W2) droplets under osmotic pressure. A) The generation of a DE droplet in the microfluidic device. The optical image shows uniformly generated DE droplets. The hydrophilic region is selectively modified with 1% polyvinyl alcohol to enhance droplet stability. B) Intact DEs in an isotonic solution. Optical image of DE droplets maintained in isotonic conditions (1× PBS), showing intact and stable droplet morphology. C) The basic mechanism of the osmotic shrinkage. The scheme shows the transportation of water from the inner aqueous phase (W1) to the outer aqueous phase (W2) through the oil layer, driven by osmotic pressure. This process involves dehydration and rehydration at the interface, leading to a reduction in the core volume of the droplets. D) Shrunken DEs in hypertonic solution. Optical image of DE droplets in hypertonic conditions (8× PBS), showing significantly reduced core volume due to osmotic shrinkage. E) The distribution of core diameters of DE droplets before and after osmotic shrinkage. The data show a significant reduction in core size due to osmotic pressure‐induced volume reduction. F) The distribution of the outer diameters of DE droplets before and after osmotic shrinkage. The results demonstrate minimal change in the overall outer diameter, indicating the stability of the oil shell during the shrinkage process. All scale bars represent 100 µm. C.V., coefficient of variation; DE, double emulsion; PBS, phosphate‐buffered saline.

As depicted in **Figure** **2**A, the microfluidic device for generating DE droplets was fabricated from a single 3‐inch wafer with a thickness of 50 µm, using single‐step photolithography and regio‐selective surface modification.^[^ ^70^ ^]^

The **polydimethylsiloxane (PDMS)** microfluidic device was then bonded to a PDMS‐coated glass substrate via plasma bonding (Figure S2A, Supporting Information). Next, we selectively modified the microfluidic channel to create two distinct regions: 1) a hydrophobic area for W/O droplet generation (channels for the inner and middle phases), and 2) a hydrophilic area coated with 1% **polyvinyl alcohol (PVA)** for W/O/W droplet formation, also referred to as DEs (Figure 2A). Fluids for the inner (aqueous), middle (oil), and outer (aqueous) phases were introduced at flow rates of 100, 200, and 400 µl h^−1^, respectively. The inner phase contained 1% (v/v) Tween‐20 and 1% (w/v) **bovine serum albumin (BSA),** and the outer phase contained 2% (v/v) Tween‐20 and 2% (w/v) Pluronic F‐127 in phosphate‐buffered saline (PBS). The middle phase was comprised of Bio‐Rad oil. Under these conditions, the device continuously produced stable DE droplets for 90 min, indicating the excellent PVA coating stability in the modified area (Figure S2B, Supporting Information). Our results demonstrated uniform DE droplet production at room temperature. Droplets were collected in an Eppendorf tube for 5 min. Subsequently, 3 µl of the collected DEs was suspended in 10 µl of the outer phase on a glass slide for microscopic imaging at 10× magnification (Figure 2B). Each droplet exhibited a well‐defined inner aqueous core surrounded by an oil shell and an outer aqueous phase. The concentric layers confirmed the formation of the DEs, whereas the uniform size distribution indicated the stability and reproducibility of the droplet generation process.

Next, we resuspended the generated DEs in hypertonic solutions to induce osmotic pressure gradients and investigated the changes in both core size and outer diameter. We hypothesized that water transport occurs through the hydrated surfactants present in the middle phase. Under osmotic pressure, the hydrophilic portion of the surfactant hydrates at the interface between the oil and phase with low salt concentration (W1), diffuses to the interface between the oil and aqueous phase with a high‐salt concentration (W2), and dehydrates there (Figure 2C). Depending on the direction of the osmotic gradient, water migrates from the inner aqueous phase (W1) to the outer aqueous phase (W2) or vice versa. Under hypertonic conditions (8× PBS), the core volume of the DEs decreased, indicating a net water efflux from W1. To ensure consistency, we used 100 µL of freshly produced DEs in 1 mL of hypertonic solution. The total volume of the droplet cores was less than 10% of the continuous‐phase volume during osmotic manipulation, therefore, any change in the overall concentration of the continuous phase was considered negligible. The observed shrinkage primarily reflected osmotic water transport through the hydrated surfactants in the oil shell, leading to core dehydration. Figure 2D shows the optical images of the DEs with core shrinkage at equilibrium after ≈60 min under hypertonic conditions. The notable decrease in the inner core volume under hypertonic conditions reflects the net water efflux from the inner aqueous (W1) to outer phase (W2), confirming that osmotic gradients can be used to modulate droplet size.

The droplet size was largely determined by the dimensions of the microfluidic orifice and the flow rates of the three phases. As expected, the DE droplets have a single core with a narrow size distribution. The average dimension of DE droplets is produced with an inner core diameter of 72.6 ± 0.8 µm **(coefficient of variation (C.V.)** ═ 1.17%) and an outer diameter of 96.5 ± 2.3 µm (C.V. ═ 2.42%) (Figure 2E,F). A high flow rate in the inner phase generated multi‐core droplets. Conversely, very low inner‐phase flow rates reduce the number of single‐core droplets, owing to insufficient dispersion in the oil phase. Notably, no core fusion was observed, indicating that each droplet acted as an independent microreactor. Based on those experiments, we determined that Q_W1_═100 µl h^−1^, Q_W2_═400 µl h^−1^, and Q_o_═200 µl h^−1^ are optimum to generate uniform, stable, single‐core DE droplets. When these droplets were resuspended in hypertonic 8× PBS, the core diameter gradually decreased, whereas the oil shell became relatively thicker (Figure 2D). Under these osmotic conditions, the core diameter decreased to 45.76 ± 0.8 µm (C.V. = 1.79%) and the outer diameter was 91.9 ± 1.5 µm (C.V. = 1.84%) (Figure 2E,F), confirming that the droplets remained stable yet exhibited significant core shrinkage.

The geometry of DE droplets is a critical design parameter that directly influences assay performance. The inner core diameter is the most crucial factor because it defines the initial reaction volume. Our key strategy, osmotic shrinkage, reduced this volume to significantly increase the local concentration of the reaction components and cleaved reporters, which is essential for enhancing the fluorescence signal and achieving a low limit of detection. The oil shell thickness was an important tradeoff: thinner shells enable faster water transport and more rapid signal enrichment, whereas shells that are too thin can compromise droplet stability and risk leakage. Finally, the outer diameter was optimized for stable droplet generation and robust performance during the high‐throughput reinjection and sorting of our microfluidic devices.

Furthermore, the W/O/W DE architecture offered significant advantages over the conventional single emulsions (W/O or O/W). First, it provided enhanced stability; the outer aqueous phase acted as a protective buffer, ensuring the structural integrity of each droplet in complex biological media, such as serum, where W/O single emulsions are notoriously unstable. Second, it enabled unique tunability. The core‐shell structure created a semipermeable barrier between the two aqueous phases, which was a prerequisite for controlling the inner core volume via osmosis. This feature, which is impossible to achieve with single emulsions, is the foundation of our on‐demand amplification‐free signal enhancement and the core innovation of our work.

### 2.3 Tunable DE Droplets

The transport of water across the droplet interface could be precisely controlled by adjusting the osmolarity of the continuous outer phase. Under hypertonic conditions, where the osmolarity of the continuous phase (W2) is higher than that of the inner aqueous core phase (W1), water efflux leads to the isotropic shrinkage of the droplet. During this process, the thickness of the oil shell increased, whereas the osmotic pressure difference gradually decreased, leading to a decay in the water flux. Eventually, the system reached isotonic equilibrium, and no further change in the core volume was observed. The extent of droplet shrinkage, and thus the final core size, was determined by the osmolarity difference between the inner (W1) and outer (W2) aqueous phases.

**Figure 3:**
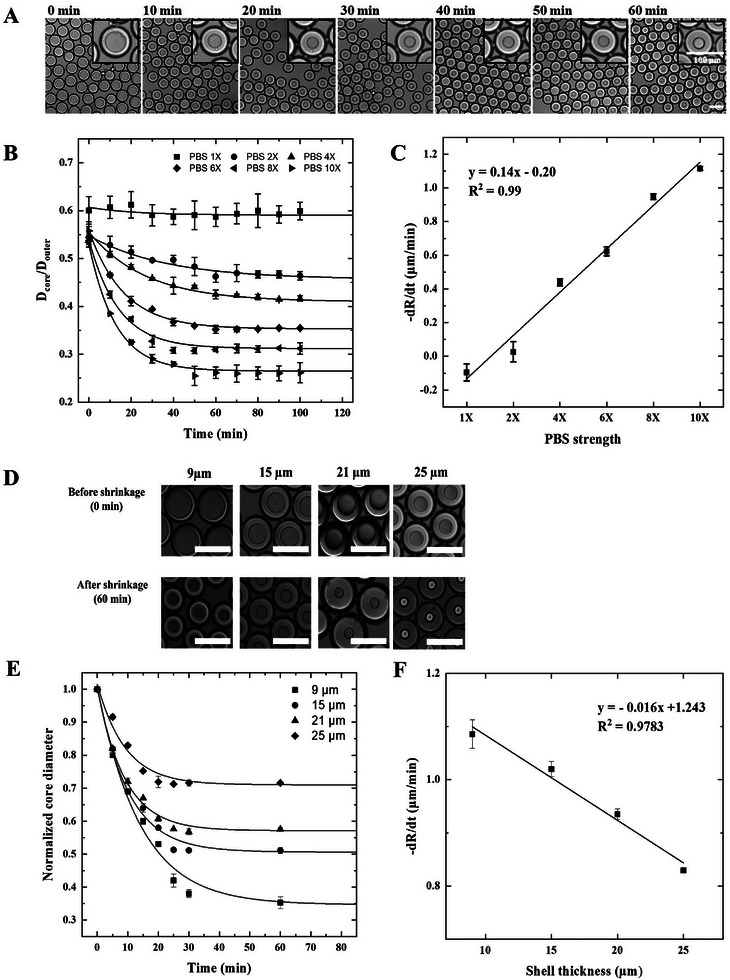
Osmotically tunable shrinkage and transport kinetics of DE droplets under varying shell thickness and osmotic conditions. A) Time‐dependent optical microscopy images of DE droplets with 1× PBS as the inner aqueous phase (W1), transferred to outer phases (W2) containing 1×, 2×, 4×, 6×, 8×, or 10× PBS. The core diameter decreases progressively under hypertonic conditions due to osmotic water efflux. B) Relative core diameter *D_rel_* (t)  =  *D_core_* (t)/*D*_*outer*_(t) as a function of time, fitted to an exponential decay model. C) Estimated characteristic timescales (τ) of core shrinkage for each W2 condition (1× to 10× PBS). D) Microfluidic control of shell thickness through tuning the flow rates of the inner aqueous (Q_W1_) and oil phase (Q_0_), while maintaining a constant outer aqueous phase flow (Q_W2_). E) Time‐lapse images of DE droplets with varying shell thicknesses (9, 15, 21, and 25 µm) exposed to 8× PBS. F) Correlation between shell thickness and core diameter reduction rate. All experimental data represent the statistical analysis of over 1000 individual droplets for each condition. DE, double emulsion; PBS, phosphate‐buffered saline.

To investigate the influence of buffer composition on droplet shrinkage kinetics, we prepared four sets of DE droplets using 1× **PBS** as the inner aqueous phase. Each set was then exposed to outer aqueous solutions containing 4×, 6×, 8×, and 10× PBS solution, respectively (**Figure** **3**A–C). All four sets of droplets exhibited progressive shrinkage of the inner aqueous core, driven by water efflux, resulting from the imposed osmotic gradient. The results show the effects of the buffer concentration of the outer phase (W2) on stability and behavior of the DEs, which can help with tailoring these systems. For example, in a system using 1× PBS as the inner phase (W1) and 8× PBS as the outer phase (W2), the core diameter decreased gradually owing to the hypertonic condition. Figure 3A shows the time‐dependent decrease in the core diameter of DE droplets prepared with 1× PBS (W1) and transferred into the 8× PBS outer phase (W2). Under hypertonic conditions, significant shrinkage was observed at 10‐min intervals over a period of 60 min, with most droplets reaching equilibrium by the end of the monitoring period. For instance, the core diameter decreased from 68 to 36 µm over 60 min. A similar shrinkage behavior was observed in droplets exposed to 4×, 6×, and 10× PBS solution in the outer phase (Figure S3, Supporting Information). This experiment demonstrated that the buffer composition in the continuous phase plays a critical role in modulating the osmotic response and volume dynamics of DE droplets, and higher concentrations of the outer phases create stronger osmotic gradients, driving more pronounced water efflux from the inner aqueous phase (W1), leading to more significant shrinkage (Figure 3B,C).

The temporal changes in the core diameter of the DE droplets relative to that of the outer shell are shown in Figure 3B. This relative diameter, denoted as *D_rel_* (t)  =  *D_core_* (t)/*D*_*outer*_(t), is evaluated for different osmolarities of the outer phase (W2) throughout the incubation period. The results show that *D_rel_* (t) rapidly decreased from its initial value *D_rel_* (0) and gradually approached a steady‐state value, *D_rel_* (∞). The time‐dependent behavior of *D_rel_* (t) is described by the following exponential decay function:

(1) $$D_{rel}\left(t\right)=\frac{D_{core}\left(t\right)}{D_{\;outer}\left(t\right)}=\left[D_{rel}\left(0\right)-D_{rel}\left(\infty \right)\right]\exp\left(-\frac{t}{\tau }\right)+D_{\mathrm{rel}}(\infty )$$

Fitting the experimental data to Equation (1) revealed that there was no significant induction time at the beginning of the shrinkage process. This result indicated that the saturation of the reverse micelles of the surfactants in the oil layer with water was short. ^[^ ^59^, ^71^, ^72^, ^73^, ^74^ ^]^ The extracted characteristic time constants (τ) for W2 conditions of 4×, 6×, 8×, and 10× PBS are 26.4, 17.8, 13.8, and 12.4 min, respectively. The reduction in the characteristic timescales also confirmed that the initial driving force for the water efflux was higher for a higher initial osmolarity. This time‐dependent change in relative diameter gradually decreased until it became nearly constant (Figure 3B). This result indicated that the salt concentration in W1 increased until it equaled the salt concentration in W2, thereby reducing the osmolarity difference between the inner core (W1) and outer phase (W2). The salt concentration ratio between the inner and outer aqueous phases was positively correlated with the rate and extent of core shrinkage, which is consistent with van't Hoff's law. ^[^ ^59^, ^75^, ^76^, ^77^ ^]^ Thus, the temporal change throughout the incubation period is predicted to cease when the osmotic pressure difference is precisely balanced by the Laplace pressure, a condition typically achieved at ≈100 min.

In this system, water passes through the middle phase (i.e., the oil shell) with limited water solubility; therefore, time is required for the inner core to reach equilibrium. As shown in Figure 3B and Figure S3B (Supporting Information), the core diameter progressively decreased under all tested PBS concentrations, indicating osmotic dehydration of the internal phase (W1). The initial rapid decrease in diameter suggests that osmotic pressure is most significant at the beginning and diminishes as equilibrium approaches. Meanwhile, the outer diameter remained largely unchanged, potentially owing to changes in the oil shell thickness or formation of hydrated surfactant structures (e.g., reverse micelles) within the oil phase (Figure S3C, Supporting Information). Despite the reduction in internal water volume, the droplets maintained excellent structural integrity under both isotonic and hypertonic conditions. This stability is crucial for downstream analyses and potential applications in which a controlled release from the core is desired.^[^ ^75^ ^]^ To quantify the effects of different osmotic gradients, we measured the water transport rates, as shown in Figure 3C. The water transport rate (slope) is defined as follows:

(2) $$\mathrm{Water}\;\mathrm{transport}\;\mathrm{rate}=-\frac{dR}{dt}$$

where “R” is the radius of the core droplet and “t” is time. Our results showed that the rate of water transport is directly proportional to the magnitude of the osmotic gradient. Therefore, the water transport rate increased as the magnitude of the osmotic gradient increased, indicating that the dehydration of the surfactant at the O/W2 interface was the controlling factor.

In addition, Figure S4 (Supporting Information) shows the reversible swelling and shrinking of the DE droplets upon repeated osmotic cycles. This behavior was fully reversible and reproducible under repeated osmotic perturbations. Although expansion does not directly improve detection sensitivity, it validates droplet stability and reversibility, thereby supporting the robustness of the DE platform. These findings demonstrate that the size of the DE droplets can be dynamically modulated after generation by tuning the osmolarity of the external environment. This osmotic responsiveness provides a versatile strategy for real‐time droplet manipulation without altering droplet composition or structure. The reversible osmosis‐driven volume change in the inner core is particularly advantageous for sensing applications where it can serve as a physical transducer of local environmental changes. Furthermore, the ability to control droplet shrinkage through osmotic pressure enables the precise regulation of encapsulated contents, offering a promising mechanism for controlled on/off release in biomedical applications, such as drug delivery or diagnostics.^[^ ^78^, ^79^, ^80^ ^]^ Collectively, these results confirm that the buffer composition plays a critical role in governing osmotic shrinkage kinetics, ultimately enabling fine‐tuned control over the core volume and function in stimuli‐responsive emulsion systems.

Furthermore, these results imply that reducing the oil‐shell thickness enhances the permeation rate by shortening the diffusion path across the barrier. To systematically investigate the effect of shell thickness on the kinetics of core shrinkage under an osmotic gradient, we generated DE droplets with shell thicknesses ranging from 5to 25 µm. Using a microfluidic approach, we precisely tuned the shell thickness by adjusting the flow rates of the inner aqueous phase (Q_W1_) and middle oil phase (Q_O_), while maintaining a constant flow rate for the outer aqueous phase (Q_W2_) (Figure 3D–F). This method provides high tunability of droplet geometry with excellent size uniformity, enabling independent control over both the core and overall droplet diameters. Although ultra‐thin‐shelled DE droplets (5and 7 µm) can be generated, some exhibited rupture during mechanical handling due to their fragility. Therefore, we selected DE droplets with shell thicknesses of 9, 15, 21, and 25 µm to investigate the relationship between shell thickness and the corresponding change in core size under a uniform osmotic pressure (Figure 3D–F).

DE droplets with varying shell thicknesses were suspended in 8× PBS (W2) for 60 min, and temporal changes in the core size were monitored (Figure 3E). Over the incubation period, the core diameter decreased to ≈65% of its initial value, resulting in core volumes significantly smaller than those of the surrounding shell layer. Optical microscopy images confirmed the presence of well‐defined core‐shell structures. In the early stages, a noticeable shrinkage in the core size was observed across all shell thicknesses. Droplets with thicker shells (21 and 25 µm) better maintained their structural integrity compared with those with thinner shells (9 and 15 µm), suggesting that increased shell thickness provides greater resistance to osmotic stress. Notably, droplets with the thinnest shell (9 µm) exhibited the most rapid and pronounced reduction in core diameter, with a steep decline observed within the first 5 to 10 min (Figure 3E). In contrast, droplets with thicker shells (15, 21, and 25 µm) showed a more gradual decrease in core size over time. The slowest shrinkage rates were observed under the thickest shell condition, highlighting the critical role of shell thickness in modulating osmotic flow and mechanical resistance. Nonetheless, all the droplets eventually reached a plateau in the core diameter, indicating an equilibrium state in which the osmotic forces were balanced by the mechanical resistance of the shell.

Overall, these findings indicate a clear trade‐off: ultra‐thin shells accelerate osmotic shrinkage but are prone to rupture, whereas thick shells enhance stability but reduce responsiveness. Therefore, we selected DE droplets with a shell thickness of ≈15 µm, which represented an optimal and practical compromise between structural stability and osmotic responsiveness, enabling reproducible and sensitive amplification‐free detection.

To gain deeper insight into the water transport mechanism, we calculated the reduction rate of the core diameter to assess the variation in water flux across the W1/O and O/W2 interfaces for different shell thicknesses. As shown in Figure 3F, a linear correlation was observed between the shell thickness and rate of core diameter reduction, indicating that the oil shell acts as a rate‐limiting barrier for water transport. These results support the hypothesis that water exchange between the two aqueous phases occurs via a hydrated‐surfactant‐mediated mechanism, in which the oil phase poses a diffusional barrier that limits transport. As the shell became thicker, the diffusion path length increased, resulting in slower water flux across the interfaces. This suggests that shell thickness plays a critical role in governing the rate‐controlling step of water transport from surfactant dehydration at the oil/water (O/W2) interface to rehydration at the water/oil (W1/O) interface.

These findings underscore the importance of shell thickness as a key design parameter in DE systems, in which precise control over core stability and release kinetics is essential. Thicker shells not only mitigate osmotic stress more effectively but also enhance mechanical robustness during microfluidic re‐injection and long‐term storage. In contrast, DE droplets with ultra‐thin shells are more prone to rupture under physical stress, highlighting the necessity for shell optimization to preserve compartmentalization and functional integrity in practical applications.

### 2.4 Enhancement of Fluorescence Intensity

**Figure 4:**
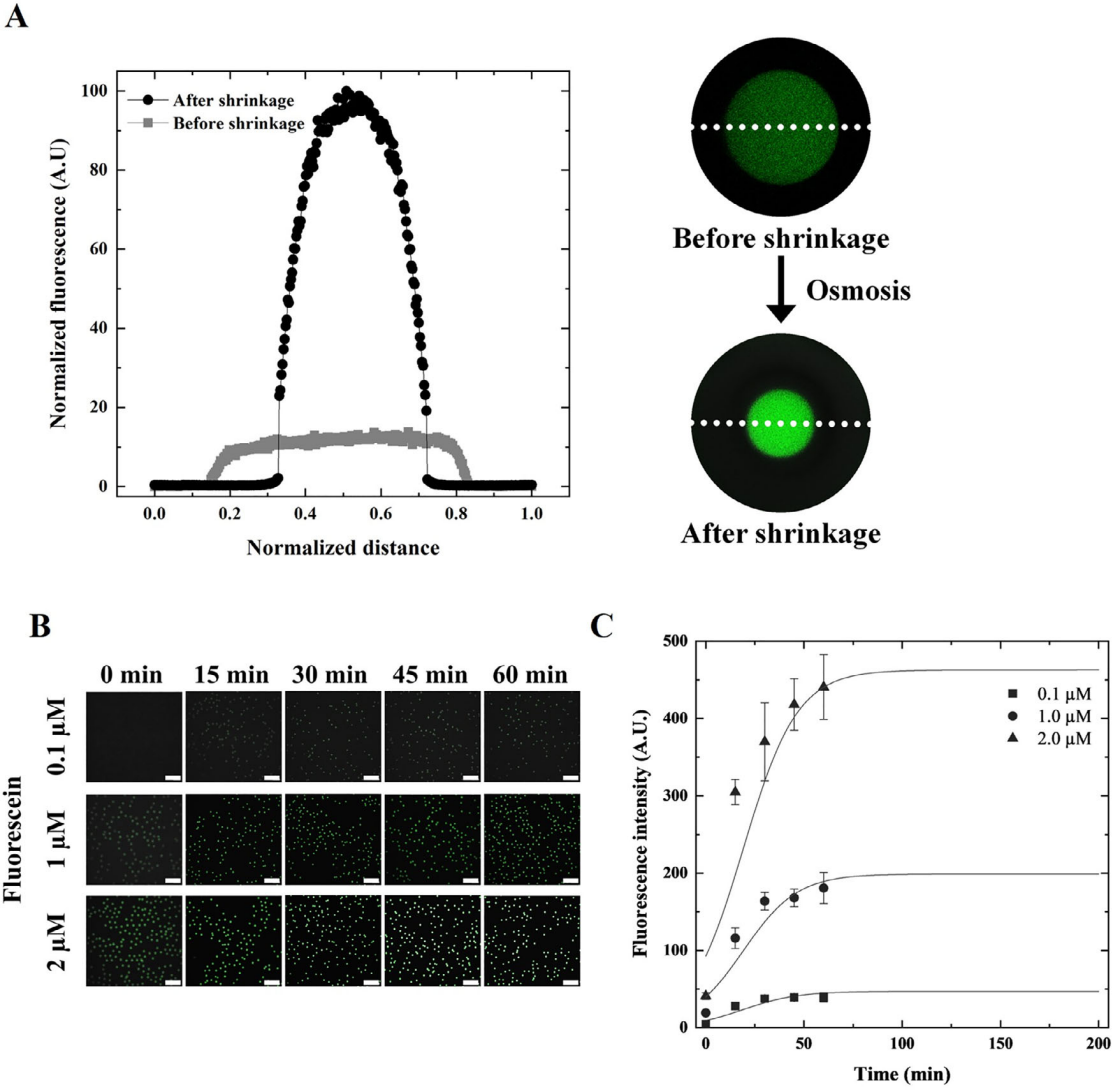
Enhancement of fluorescence intensity in DE droplets via osmotic shrinkage. A) Representative line profiles of fluorescence intensity in DE droplets before and after osmotic shrinkage. DE droplets encapsulating fluorescein (1.0 µM) exhibit a clear increase in fluorescence intensity following exposure to a hypertonic solution (8× PBS). B) Time‐dependent fluorescence intensity measurements for DE droplets containing three different initial fluorescein concentrations (0.1, 1.0, and 2.0 µM). Fluorescence signals are monitored at 15‐min intervals over 60 min after resuspension in 8× PBS. C) Quantification of fluorescence enhancement. The fluorescence intensity increased by 8.6‐, 9.4‐, and 10.8‐fold for initial fluorescein concentrations of 0.1, 1.0, and 2.0 µM, respectively. All experimental data represent the statistical analysis of over 1000 individual droplets for each condition. DE, double emulsion; PBS, phosphate‐buffered saline.

In this study, we encapsulated fluorescein molecules in the inner aqueous phase of DE droplets to investigate how osmotic shrinkage enhances the fluorescence intensity. This approach enables direct visualization of fluorescence signal amplification resulting from osmotic pressure‐induced volume reduction. All imaging procedures were performed in a dark environment to minimize photobleaching. As a proof of concept, DE droplets are prepared with three different initial fluorescein concentrations (0.1, 1.0, and 2.0 µM) and resuspended in a hypertonic solution (8× PBS). Fluorescence signals were monitored at 15‐min intervals for up to 60 min following osmotic exposure (**Figure** **4**).

As shown in Figure 4A, the line profile of the fluorescence intensity markedly increased after osmotic shrinkage, indicating that water transport through the semipermeable oil layer concentrated the fluorescein within the core. In addition, the spatial fluorescence distribution before and after shrinkage demonstrated not only signal enhancement, but also increased localization of the fluorescent signal within the center of the droplet. This finding supports the idea that volume reduction leads to a radial concentration of solutes, resulting in a steep intensity gradient, which is evidence of molecular confinement.

For droplets encapsulating 0.1 µM fluorescein, fluorescence is initially undetectable at 0 min (Figure 4B). However, within 15 min of exposure to the hypertonic solution, the fluorescence signal became visible owing to osmotic volume reduction and the resulting enrichment of fluorescent molecules within the confined core. Similarly, droplets containing 1.0 and 2.0 µM fluorescein showed a progressive increase in fluorescence intensity over time, eventually reaching a plateau as osmotic equilibrium is established (Figure 4B,C). The temporal progression of fluorescence signals shows that even at the lowest initial concentration (0.1 µM), osmotic shrinkage can elevate the signal above the detection threshold. This method enables signal rescue from near‐background levels, which is particularly valuable for low‐copy‐number molecular diagnostics. As shown in Figure 4C, fluorescence intensity increased over time for all initial fluorescein concentrations, with higher starting concentrations (1.0 and 2.0 µM) resulting in greater absolute signal enhancement. These results indicate that the fluorescence signal amplification scaled with the initial solute concentration and no apparent molecular crowding, self‐quenching, or detection saturation occurred within the tested range. This time‐dependent signal enhancement is attributed to water‐efflux‐induced core shrinkage, which concentrates the encapsulated fluorophores. After 60 min, the fluorescence intensity increased 8.6‐, 9.4‐, and 10.8‐fold for the initial fluorescein concentrations of 0.1, 1.0, and 2.0 µM, respectively. These results demonstrate the effectiveness of osmotic shrinkage in amplifying the fluorescence signals within DE droplets and reveal a nonlinear relationship between the initial fluorescein concentration and signal enhancement, suggesting a combined effect of concentration and spatial confinement. Notably, the fold increase was consistent across concentrations, indicating that the degree of enrichment was largely governed by volume shrinkage rather than absolute concentration. This finding supports the hypothesis that osmotic shrinkage is a universal amplification mechanism.

Therefore, DE droplets with osmotic shrinkage capability have a strong potential for signal amplification in applications, such as the selection or screening of low‐abundance target molecules.

### 2.5 Detection of the *hEPO* Gene in the Doping Test

CRISPR‐Cas12a is an RNA‐guided endonuclease that binds to and cleaves target DNA and exhibits trans‐cleavage activity against non‐specific single‐stranded DNA (ssDNA).^[^ ^81^ ^]^ By incorporating a fluorescence/quencher‐labeled ssDNA reporter, we used targeted dsDNA‐triggered fluorescence activation to detect the *hEPO* gene relevant to gene doping.

To select the osmotic conditions for all subsequent detection experiments, we systematically evaluated droplet performance under different buffer concentrations (Figures 3 and 4). Although low osmotic gradients (e.g., 2× or 4× PBS) resulted in only modest fluorescence enhancement, excessive osmotic stress in 10× PBS led to structural instability and droplet rupture. Among the tested conditions, 8× PBS provided the most favorable balance, yielding strong and reproducible fluorescence amplification while preserving droplet integrity. Therefore, 8× PBS was used as the standard osmotic solution.

To demonstrate the integration of the CRISPR‐Cas12a system with a DE platform, the reaction mixture containing the *hEPO* gene (5.8 fmol µL^−1^) was encapsulated in DE droplets. Initially, the flow rates were optimized to ensure stable, monodisperse DE generation. The phase diagram illustrates a broad range of stable droplet formation regimes achieved under a constant outer phase flow (200 µL h^−1^) (Figure S5, Supporting Information). We produced over 20 000 DE droplets with core volumes of ≈0.3 nL. The droplets, suspended in isotonic 1× tris‐buffered saline (TBS), were incubated at 37 °C for 60 min.

**Figure 5:**
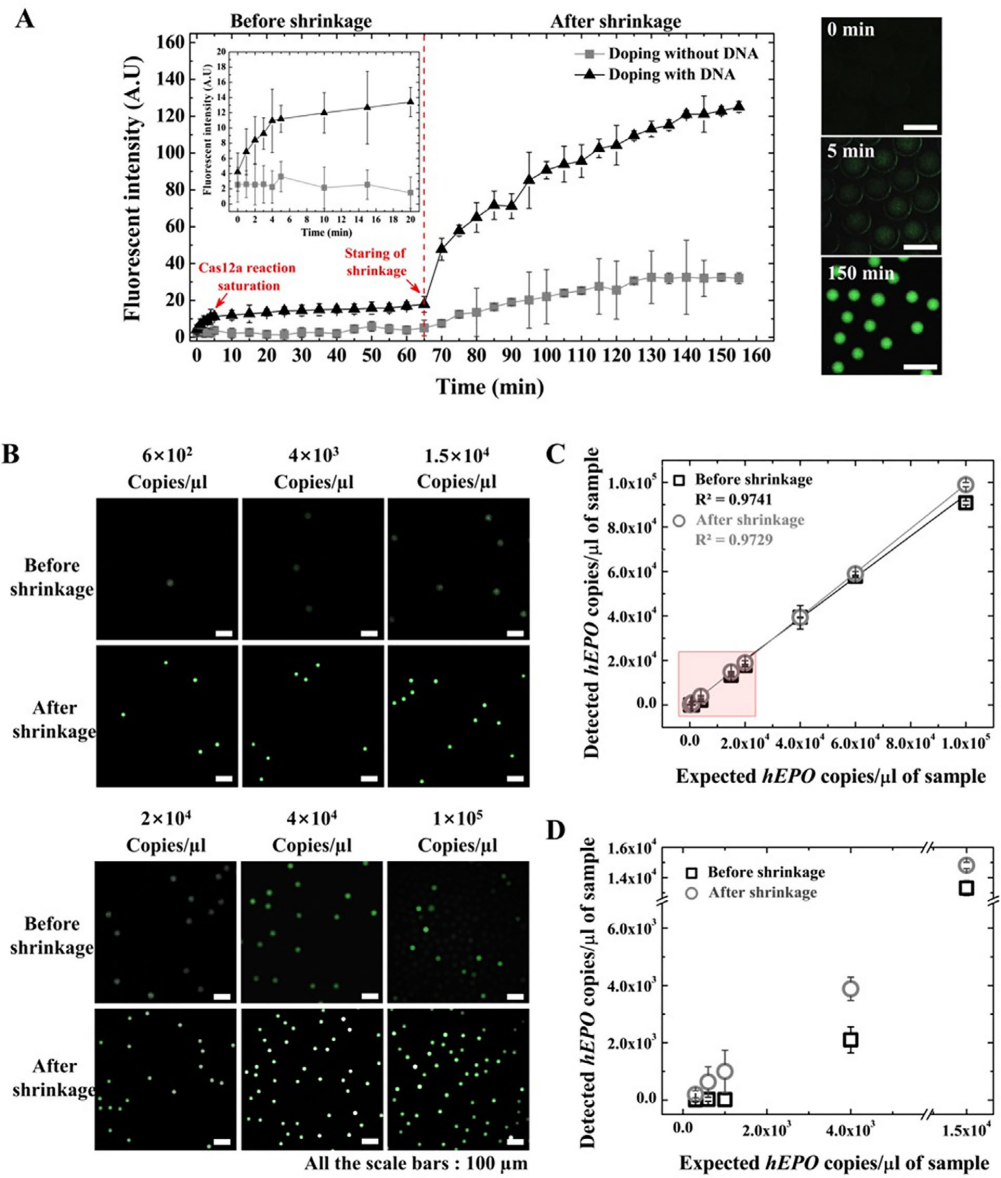
Detection of the transfected *hEPO* gene using shrinkable DE droplets. A) Comparative analysis of fluorescence intensity in DE droplets with and without *hEPO* gene doping. The inset shows a rapid increase in fluorescence within the first 5 min. Fluorescence remains stable for up to 60 min under isotonic conditions (1× buffer). Subsequent osmotic shrinkage is induced by replacing the external buffer with an 8× buffer solution. Time‐lapse fluorescence images (right) demonstrate the progressive signal amplification driven by Cas12a‐mediated trans‐cleavage of 5‐FAM‐3‐BHQ1‐labeled ssDNA reporters. Scale bars: 100 µm. B) Fluorescence microscopy images of DE droplets containing varying concentrations of the *hEPO* gene before and after osmotic shrinkage. Scale bars: 100 µm. C) Quantitative analysis showing the linear correlation between expected (X‐axis) and detected (Y‐axis) *hEPO* gene copy numbers per µL, calculated from positive droplet counts via Poisson distribution. The analysis includes six target concentrations (as in B) plus the 0 copies µL^−1^ control, for a total of seven points. The red box highlights the lower concentration range (300–4000 copies µL^−1^). D) Enlarged view of the red box from panel (C), comparing the limit of detection before and after shrinkage. All data are presented as the mean ± standard deviation of three independent experiments (n = 3). For each experiment, quantitative data were derived from the analysis of over 20 000 individual droplets per condition. DE, double emulsion.

As shown in the inset of **Figure** **5**A, Cas12a trans‐cleavage rapidly increased fluorescence intensity within the first 5 min and then plateaued, indicating early reaction saturation. Subsequently, the DE droplets were transferred to hypertonic 8× TBS buffer. Upon osmotic shrinkage, the fluorescence intensity significantly increased (≈tenfold) because of the concentration of cleaved reporters in the reduced core volume (Figure 5A). As a negative control, a blank reaction (CRISPR‐Cas12a mixture lacking the target DNA) was tested under identical conditions. No significant fluorescence was observed before shrinkage; however, a slight increase occurred after osmotic compression, likely due to background signal concentration. This emphasized the importance of using blank controls to define the threshold for reporter‐associated background signals, which is critical for accurate sample quantification.

To demonstrate the enhanced sensitivity of our platform for non‐amplified gene detection, we encapsulated the *hEPO* gene at concentrations ranging from 300 to 1×10⁵ copies µL^−1^ within DE droplets. As shown in Figure 5B, osmotic shrinkage transformed undetectable signals at lower gene concentrations into clearly detectable fluorescence signals. The CRISPR‐Cas12a reaction triggered an initial increase in fluorescence within the first 5 min, owing to ssDNA reporter cleavage. This was followed by osmotic shrinkage over the subsequent 30 min, which further concentrated the signal. This two‐step process, the initial trans‐cleavage reaction and subsequent volume reduction, enabled rapid and highly sensitive detection of the *hEPO* gene within 35 min.

Applying a digital droplet analysis approach across ≈20 000 individual droplets, we observed a clear decrease in the number of positive droplets with decreasing *hEPO* gene concentrations (Figure 5B). This confirms that our analytical system reliably reflects the target gene abundance. Quantitative analysis using Poisson statistics revealed a strong linear correlation (R^2^ = 0.97) between the expected and observed *hEPO* concentrations from 600 to 1×10⁵ copies µL^−1^ (Figure 5C).

To better visualize the detection sensitivity at lower concentrations, the red box in Figure 5C highlights the low concentration range (300 to 15 000 copies µL^−1^), which is magnified and presented separately in Figure 5D. This demonstrates the 25‐fold improvement in detection limit upon droplet shrinkage, allowing quantification down to 600 copies µL^−1^ after osmotic shrinkage, compared to 1.5×10⁴ copies µL^−1^ without shrinkage. This enhancement results from a reduction in the core volume, which concentrates both the target gene and cleaved fluorescent reporters within the confined droplet. At 300 copies µL^−1^, detection efficiency was less than expected, possibly attributable to stochastic loading or loss of target molecules, limitations commonly observed in conventional digital assays, such as ddPCR, ddRPA, and ddLAMP.^[^ ^82^ ^]^

Notably, the upper limit of our digital platform was constrained by the statistical principles of the Poisson model, which is distinct from the signal saturation observed in analog assays. This statistical constraint occurs at concentrations ≥ 1×10⁵ copies µL^−1^, where the fraction of positive droplets exceeds ≈95% and the number of negative droplets becomes too low for reliable calculation. The effective dynamic range reflects an intrinsic limitation of digital quantification, rather than Cas12a enzymatic activity. Nevertheless, the principal advantage of our platform is that it can achieve ultrasensitive quantification in a low‐concentration regime, where conventional amplification‐free methods face significant challenges.

### 2.6 Validation in Serum‐Spiked Sample

**Figure 6:**
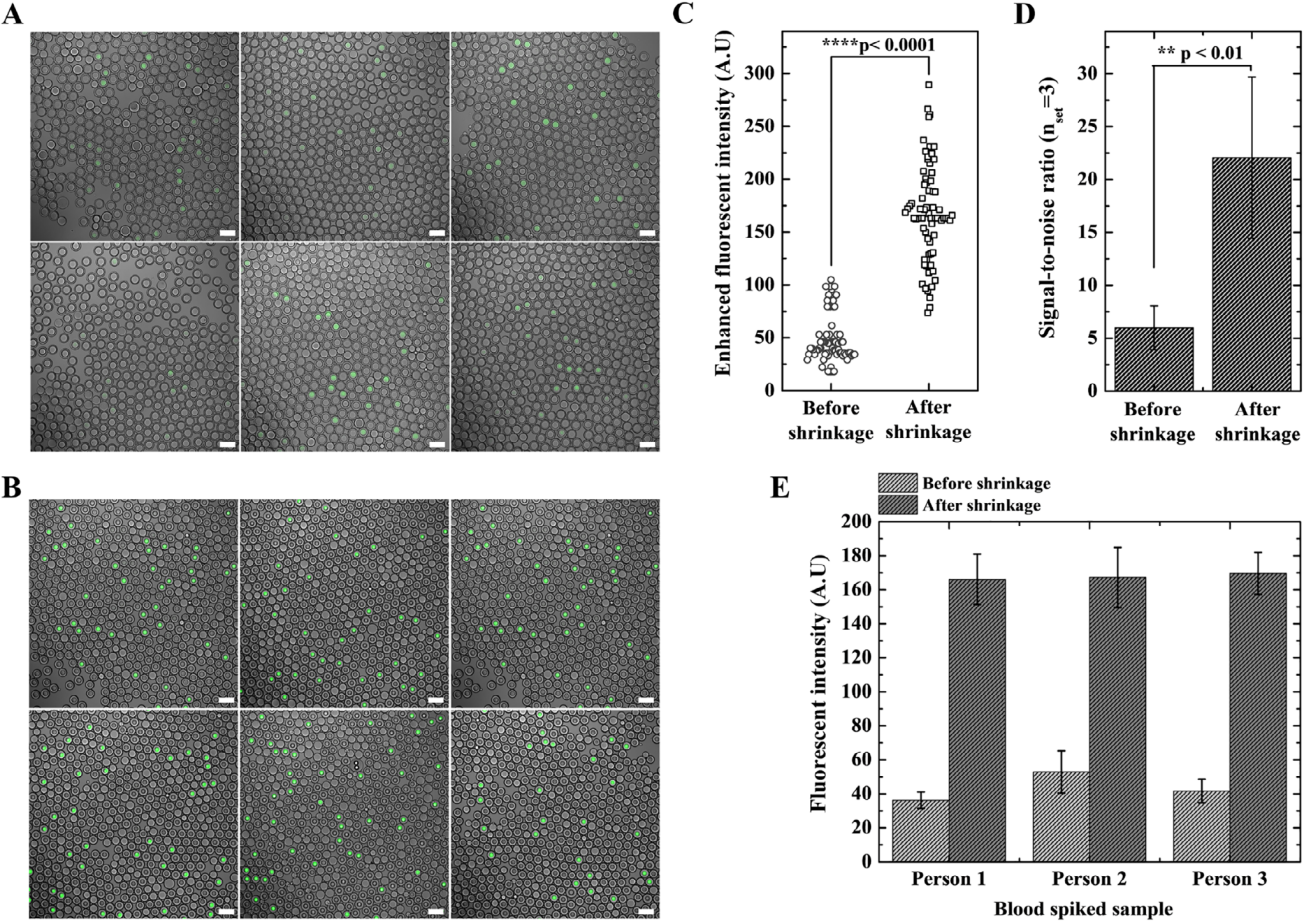
Validation of shrinkable DE droplet‐based detection in *hEPO* gene‐spiked blood serum samples. A) Merged optical‐fluorescence images showing DE droplets encapsulating a mixture of 600 copies µL^−1^ *hEPO*‐spiked blood serum and CRISPR‐Cas12a reaction reagents before osmotic shrinkage. The DE droplets contain an inner aqueous phase (1× TBS buffer) and are suspended in an outer 1× TBS buffer phase. B) Merged optical‐fluorescence images of DE droplets after osmotic shrinkage. Initially suspended in 1× TBS, the droplets are transferred to an outer 8× TBS buffer phase and incubated for 30 min, resulting in a significant increase in fluorescence due to the reduction in core volume. In panels A and B, the six images represent different microscopic regions selected as representative fields from the same experiment. C) Quantification of fluorescence intensity in individual positive droplets before and after shrinkage. A paired Student's t‐test confirms a statistically significant increase in fluorescence intensity after shrinkage. D) Signal‐to‐noise ratio (SNR), calculated as the average intensity of positive droplets divided by that of negative droplets (n=758). The SNR is significantly enhanced after shrinkage, indicating improved detection clarity. Error bars represent the standard deviation from three independent experiments. E) Assessment of batch‐to‐batch variation across blood serum samples from three individuals, each spiked with 600 copies µL^−1^ of the *hEPO* gene. Fluorescence intensity is significantly increased after shrinkage, and low variability confirms the reproducibility of the platform. Error bars correspond to the standard deviation from n = 3 independent blood serum samples (biological replicates). All scale bars represent 100 µm. DE, double emulsion: TBS, Tris‐buffered saline.

Given that the presence of a complex biological matrix can alter reaction kinetics and impact detection accuracy, we validated our platform using three blood serum samples spiked with the *hEPO* gene. Each sample was prepared by mixing serum containing 600 copies µL^−1^ of the *hEPO* gene with the CRISPR‐Cas12a reagent, resulting in a final serum concentration of 5%. The mixtures were then encapsulated into DE droplets and incubated at 37 °C for 5 min. Optical‐fluorescence composite images of the droplets are shown in **Figure** **6**A. After resuspension in 8× buffer, osmotic shrinkage led to significant enrichment of fluorescent signals (Figure 6B). The droplets exhibited a consistent ≈tenfold volume reduction accompanied by a notable increase in fluorescence intensity. This allowed for a greater number of visible positive events, comparable to those observed in the buffer‐only controls, indicating that serum had minimal inhibitory effects on the assay. Slightly elevated background fluorescence was observed in serum‐containing samples, likely due to the non‐specific adsorption of serum proteins.

Statistical analysis using a paired Student's t‐test revealed a highly significant enhancement in fluorescence intensity after shrinkage (p < 0.0001; Figure 6C). We also evaluated the SNR, which is defined as the average intensity of the positive droplets divided by that of the negative droplets (Figure 6D). Among the 758 individual droplets analyzed, the SNR was significantly higher after shrinkage, reflecting improved detection clarity. In contrast, the pre‐shrinkage droplets showed lower SNR values, suggesting signal interference from the background noise. This difference was statistically significant (p < 0.01).

Finally, to assess the reproducibility of the platform, we analyzed blood serum samples from three different individuals spiked with 600 copies µL^−1^ of the *hEPO* gene. Our platform demonstrates high reproducibility under these conditions. For batch‐to‐batch variability, the resulting fluorescence enhancement was highly consistent with a C.V. of < 10% (Figure 6E; Figure S6, Supporting Information). Furthermore, the osmotic shrinkage kinetics were robust across a wide range of environmental conditions (1× to 10× PBS), showing predictable and repeatable volume reductions (Figure 3). The structural integrity and reversibility of this process were confirmed through repeated osmotic cycles (Figure S4, Supporting Information). This demonstrates that robustness in complex biological matrices represents a key practical advantage over conventional methods, which we confirmed by direct comparison with qPCR.

Although qPCR detected targets effectively in pure water (Figure S8, Supporting Information), its performance was substantially compromised in blood‐derived samples owing to matrix interference, necessitating a time‐consuming and loss‐prone nucleic acid extraction step. This effectively limits the sensitivity of qPCR in biological samples to ≈1500 copies,^[^ ^83^ ^]^ whereas our extraction‐free platform achieved a superior quantification limit of 600 copies. Moreover, our platform provided results within 1 h, which is a significant improvement over the >2 h required for a complete qPCR workflow. Collectively, these results confirm that our osmotically tunable platform is not only robust and reproducible but also offers a faster, simpler, and more sensitive solution for gene doping detection in real‐world samples compared to the gold standard. A comparative summary is presented in Table S1 (Supporting Information).

## 3 Conclusion

The unregulated use of non‐therapeutic gene therapy by athletes poses substantial health risks and ethical challenges, underscoring the critical need for detection platforms that are sensitive, rapid, and sufficiently robust to overcome the complexities of conventional methods. In this study, we developed an innovative, amplification‐free CRISPR/Cas12a‐based diagnostic system. This platform was uniquely integrated with osmotically shrinkable DE droplets by using their inherent advantages as discrete microreactors.

Our system achieved substantial signal amplification through precisely controlled droplet shrinkage, thereby effectively enhancing the concentration of target molecules without the need for nucleic acid amplification. Our results demonstrate that under hypertonic conditions, DE droplets undergo a precise tenfold reduction in core volume, leading to an equivalent increase in the fluorescence signal. Furthermore, the CRISPR‐Cas12a system achieved maximum cleavage activity within 5 min, enabling ultra‐rapid, single‐step target recognition. This detection strategy exhibits excellent reproducibility and minimal matrix interference when applied to *hEPO*‐spiked human serum samples, strongly supporting its utility in real‐world anti‐doping scenarios.

To our knowledge, this is the first demonstration of a DE‐based CRISPR diagnostic system that synergistically combines amplification‐free detection with osmotic signal enrichment in a scalable microfluidic format. The versatility, portability, and ability of the platform to operate without complex instrumentation make it broadly applicable across diverse fields, including point‐of‐care gene‐doping surveillance, forensic genetics, biosecurity, and resource‐limited diagnostics. Taken together, these findings not only validate the technical robustness of our novel platform but also introduce a groundbreaking strategy for digital diagnostics by seamlessly integrating osmotic signal control with CRISPR‐based molecular recognition. This innovative approach paves the way for the development of next‐generation nucleic acid‐testing systems that are simple, highly sensitive, and readily deployable in the field.

## 4 Experimental Section

### Experimental Material

Photoresists (SU8 3005 and 3025) and PDMS used for device fabrication were purchased from Microchem (MA, USA) and Dow Corning (MI, USA), respectively. The oil used for making emulsion droplets was acquired from Bio‐Rad (MN, USA). Moreover, 1× and 10× PBS were obtained from Thermo Fisher Scientific (MA, USA). Fluorescein sodium salt, Tween 20, BSA, and Pluronic‐127 were purchased from Sigma‐Aldrich (MO, USA). Droplet generation oil was supplied by Bio‐Rad. The crRNAs were purchased from Bioneer (Daejeon, Korea). Primers, dual‐labeled oligo (5ʹFAM, 3ʹBHQ1) for the fluorescence reporter and gene synthesis with sub‐cloning service were purchased from Macrogen (Seoul, Korea). Luria‐Bertani (LB) and terrific broth (TB) were purchased from Difco (MI, USA). Ni‐NTA agarose was purchased from Qiagen (Hilden, Germany). Dithiothreitol (DTT), TritonX‐100, and tris (hydroxymethyl)aminomethane were purchased from Sigma‐Aldrich.

### Microfluidic Device Fabrication

The microfluidic device was fabricated using conventional photolithography and soft lithography.^[^ ^71^ ^]^ The microfluidic device wafer was manufactured using photolithography with UV exposure on a negative photoresist using a Mask Aligner (MDA‐400 M) on a 3″ silicon wafer (PI‐KEM‐WAFER‐SILI‐0004W25). SU‐8 3050 was spin‐coated onto the wafer at 2789 rpm for 30 s to reach the desired thickness of 50 µm. A master was developed using an SU‐8 developer and fluorosilanized it to prevent adhesion to the PDMS (Sylgard 184, Dow Corning) during soft lithography. For replica molding, the PDMS base and curing agent (10:1 ratio) were thoroughly mixed, poured into the mold, degassed in a vacuum chamber, and finally cured at 65 °C for 12 h. The cured PDMS was then carefully peeled off the master mold, and the inlet holes were punched and aligned over the PDMS‐coated glass substrate by plasma activation of both surfaces using a Plasma Cleaner (PDC‐002 (230 V); Harrick Plasma). Before the experiment, the microfluidic device was degassed and connected to tubing.

### Surface Treatment Process for the Selective Hydrophilic Modification of the PDMS Channel

Plasma treatment using O_2_ gas was used to render the surface hydrophilic, followed by plasma bonding with a PDMS‐coated glass slide.^[^ ^70^ ^]^ The device was then incubated at 100 °C for 1 h. Next, surface modification was performed using a 1% PVA solution. This PVA solution was manually infused into the desired area of the PDMS microchannel block, while air was blown in the opposite direction to maintain and form an air‐PVA interface. This status was maintained for 2 min to induce the stable adsorption of PVA onto the PDMS microchannel. The PVA solution was then flushed with a strong airflow to prevent unintended channel clogging. This procedure was repeated three times. The final step involved a careful microscopic examination of the microchannel and baking the device at 120 °C for 15 min to ensure stability.

### Wetting Dynamics Over Time

The wetting progression was monitored within the channels of the PDMS device for more than 90 min. Each time frame captured the aqueous‐phase wetting point, indicating the effectiveness of the surface treatment in controlling the hydrophilic properties of the PDMS microchannel.^[^ ^70^ ^]^ Starting from 0 min and continuing at 10‐min intervals, Figure S2B (Supporting Information) demonstrates the gradual wetting behavior critical for applications, such as droplet formation and microfluidic assays.

### Production of Cas12a

*Cas12a* (*AsCpf1*) containing a C‐terminal 6×His‐tag gene was cloned into the pET28b vector using an In‐fusion cloning kit according to the manufacturer's instructions. *Escherichia coli* BL21 (DE3) cells were transformed by cloning the pET28b::Cas12a plasmid and cultured on LB agar plates with kanamycin. A single colony was inoculated into 2 mL of LB with kanamycin, and an overnight culture (0.5 mL) was added to 50 mL of fresh TB. The culture was then incubated at 37 °C until the optical density (OD600) reached 0.6. Protein expression was induced by adding 1 mM IPTG. Next, cells cultured were harvested at 18 °C for 16 h using centrifugation and resuspended them in 5 mL of lysis buffer composed of 50 mM Tris‐HCl, 500 mM NaCl, pH 8.0. After ultrasonication and centrifugation, the cell lysate was incubated with the Ni‐NTA agarose resin. The resin was washed with 10 mL of a three‐wash buffer (10–30 mM imidazole gradient in lysis buffer, 10 mM step size) and eluted with 3 mL of elution buffer (400 mM imidazole in lysis buffer). The eluent buffer was replaced with 2× TBS containing 1 mM of DTT (1×TBS: 50 mM Tris‐HCl, 140 mM NaCl, pH 7.4) using a 100‐kDa ultrafiltration device. Purified Cas12a was used for 10% sodium dodecyl sulfate‐polyacrylamide gel electrophoresis and band images were analyzed using ImageJ after Coomassie blue staining. Final buffer composition was 50 mM Tris‐HCl, 140 mM NaCl, 1 mM DTT, 50 v/v% glycerol, pH 7.4, and the stock was stored at −20 °C until further use.

### Design of Target‐Specific crRNAs

The sequences of the target genes used in this study originated from the verified GenBank database and crRNAs were prepared based on this data [*EPO* (GenBank NM_000799.4)]. Two main criteria were considered for crRNA design: The 5′TTTV sequence was used as a PAM, and the crRNA sequence following the PAM should cover the exon‐exon junction of the target exogenous gene. The designed crRNA target sequence was synthesized with a 5ʹ scaffold for AsCpf1 (5ʹUAAUUUCUACUCUUGUAGAUAGCACAGCCCGUCGUGAUAU3ʹ; the underlined sequence was the target sequence). The copy number of the exogenous gene was calculated based on the molecular weight of plasmid pCMV:*EPO* (5460 bp, 3 374 142.31 g mol^−1^).

### Digital Detection and Poisson Analysis of the hEPO Gene

A microfluidic sorter device was used for the high‐throughput screening and quantitative analysis of DE droplets (Figure S7, Supporting Information). This device featured two inlets: one for reinjecting the DE droplet mixture suspended in the continuous aqueous phase and the other for introducing a spacing fluid. These streams merge at a sample junction that enables the spatial separation of individual droplets before they pass through a defined laser detection zone, thereby minimizing the signal overlap during analysis. To detect the fluorescence signals, a laser beam was directed through a multi‐edge dichroic beam splitter and an inverted fluorescence microscope. Inside the microscope, the beam was reflected upward by a planar mirror and focused through an objective lens to form a laser spot across the microfluidic channel. As the DE droplets flowed past this spot, the fluorescent emission from each droplet was collected through the same optical path and directed to a photomultiplier tube (PMT) via a dichroic mirror. A LabVIEW‐based acquisition system with a high‐speed camera monitored the emitted fluorescence in real time, allowing discrimination between positive and negative droplets based on predefined fluorescence intensity thresholds. The DE droplets with fluorescence intensities exceeding the threshold were classified as positive, indicating successful encapsulation and reaction with the *hEPO* target gene. Positive droplets were defined as those exhibiting fluorescence intensities exceeding the threshold established from a no‐target control (NTC) and were calculated as the mean (NTC) + 3×SD(NTC). The counts of positive (*k*) and total droplets (n) were used for the Poisson statistical analysis. The absolute concentration of the *hEPO* gene (λ, mean copy number per droplet) was estimated using the Poisson equation:

(3) $$\lambda =-\ln\left(1-\frac{k}{n}\right)$$

The calculated λ was converted into absolute concentration (copies/µL) using the known droplet volume. The experiments were conducted in triplicate to ensure reproducibility. The linear relationship between measured λ values and known *hEPO* concentrations was plotted to assess detection accuracy and dynamic range.

### qPCR

For qPCR analysis of hEPO, a 20‐µL reaction mixture was prepared using 10 µL of iQ SYBR Green Supermix, 5 µL of a primer mix (final concentration of 500 nM), and 1 µL of EPO standard DNA. The forward and reverse primer (RP) sequences were 5ʹ‐ATGGGGGTGCACGAATGTC‐3ʹ and 5ʹ‐AGACTCGGAAGAGTTTGCGG‐3ʹ, respectively. Amplification was performed using LightCycler 480 II (Roche, Indianapolis, IN, USA). The cycling conditions were an initial denaturation at 95 °C for 10 min, followed by 40 cycles of 95 °C for 20 s and 63 °C for 45 s. All reactions were conducted in triplicate (n═3). Fluorescence was measured at an emission wavelength of 510 nm and an excitation wavelength of 465 nm at the end of the extension step of each cycle. The primers used for qPCR were purchased from Macrogen (Seoul, Republic of Korea). iQ SYBR Green Supermix was purchased from Bio‐Rad.

### Droplet Imaging and Analysis

The droplets were collected on EVE cell counting slides (NanoEntek; Cat no.: ATG‐EVS‐050) for imaging. An inverted fluorescence microscope (TE2000; Nikon, Japan) equipped with a CCD camera (Coolsnap; Photometrics, Tucson, AZ) was used to observe microdroplets. Image analysis of the microfluidic device and droplets was performed using ImageJ (National Institute of Health, Maryland) and Image‐Pro (Media Cybernetics, City, MD) software programs. The fluorescent images of nearly 1000 droplets per image were first converted to binary masks based on the intensity threshold using ImageJ software. The boundaries of the resulting droplet were filled using the function “watershed”. After adjusting the appropriate scale of the image, droplets were analyzed using the function “Analyze particle”. The obtained information, such as the droplet area and diameter, was used for volume calculation. Similarly, the intensity of each droplet was calculated.

### High‐Speed Camera Imaging and Data Analysis

High‐speed camera imaging was conducted using a Phantom Miro eX2 camera with a resolution of 640 × 480 pixels and a frame rate of 1246 fps. An LED light source provided illumination, and the data was analyzed using the Sapphire 561‐300 LPX CDRH laser, with a 561.00 nm wavelength and 0.70 mm beam diameter. The camera was connected to a microscope and linked to a standard analog video monitor (PAL or NTSC) for real‐time image monitoring. Phantom software was used to save videos in formats such as AVI, and individual frames were saved as TIFF images. Fluorescence was detected using two H10722‐20 photomultipliers (Hamamatsu, Japan).Statistical Analysis

All experiments were performed at least in triplicate, and data were presented as the mean ± standard deviation (SD). Statistical significance for quantitative comparisons (e.g., fluorescence intensity, droplet size, or SNR) was determined using a paired two‐tailed Student's t‐test unless otherwise noted. Statistical significance was set at p < 0.05. No data transformations or outlier exclusions were performed. No multiple comparisons or post‐hoc adjustments were performed unless otherwise specified. All statistical analyses and curve fitting were performed using SPSS, Microsoft Excel, and OriginPro 8.1.

### Ethics

Whole blood samples used in the experiment were collected from the Korea Anti‐Doping Agency, and only anonymous samples approved for research purposes were used. In addition, this research was conducted with the approval of the Institutional Review Board of Korea Institute of Science and Technology (approval number: KIST‐202304‐BR‐006) according to the research management of human materials. Serum samples were obtained from whole blood samples after centrifugation at 1500×g for 15 min, and stored at –4 °C until further use.

## Conflict of Interest

The authors declare no conflict of interest.

## Supporting information



Supporting Information

## Data Availability

The data that support the findings of this study are available from the corresponding author upon reasonable request.
