# Supplementary material for: Osmotically Tunable Microdroplets Enable Amplification‐Free CRISPR Detection of Gene Doping
Source: Adv Sci (Weinh). 2025 Oct 14;12(48):e15861. doi: 10.1002/advs.202515861 (PMC12752553; doi:10.1002/advs.202515861)
Supplement: Supplementary file 1 — Supporting Information [file ADVS-12-e15861-s001.docx]

Supporting Information

Osmotically Tunable Microdroplets Enable Amplification-Free CRISPR Detection of Gene Doping

*Jihun Han^+, [a]^, Reya Ganguly^+, [a]^, Joon-Yeop Yi^+, [b, c]^, Hyewon Yun ^[a]^, So-Yeon Jung ^[a]^,*

*Changmin Sung ^[c],^ *, and Chang-Soo Lee ^[a],^ **

[a] J. Han, R.Ganguly, H. Yun, S.Y. Jung, C.S. Lee
Department of Chemical Engineering and Applied Chemistry,
Chungnam National University
Yuseong-gu, Daejeon 34134, Republic of Korea
E-mail: rhadum@cnu.ac.kr

[b] J.Y. Yi
Interdisciplinary Program of Bioengineering,
Seoul National University,

Seoul, 08826, Republic of Korea

[c] J.Y. Yi, C. Sung
Doping Control Center, Korea Institute of Science and Technology,

Seoul, 02792, Republic of Korea

E-mail: scm@kist.re.kr

[+] Authors contributed equally to this work

[*] Corresponding authors


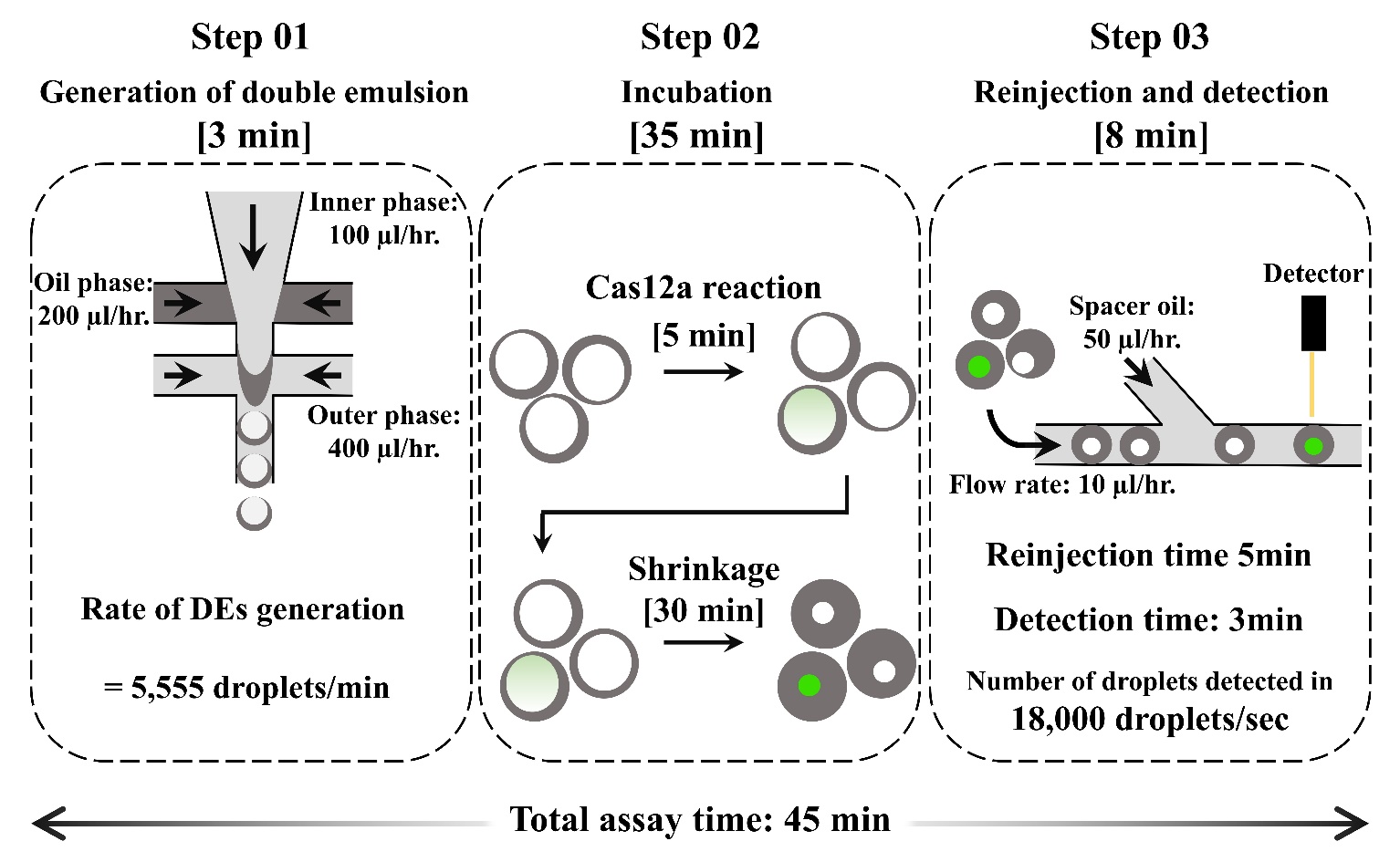


**Figure S1**. Total process of double emulsion (DE)-based gene doping. The process consists of three key steps: (1) microfluidic generation of DE droplets, (2) off-chip incubation for Cas12a reaction and osmotic shrinkage, and (3) high-throughput reinjection and fluorescence-based detection using a microfluidic system.


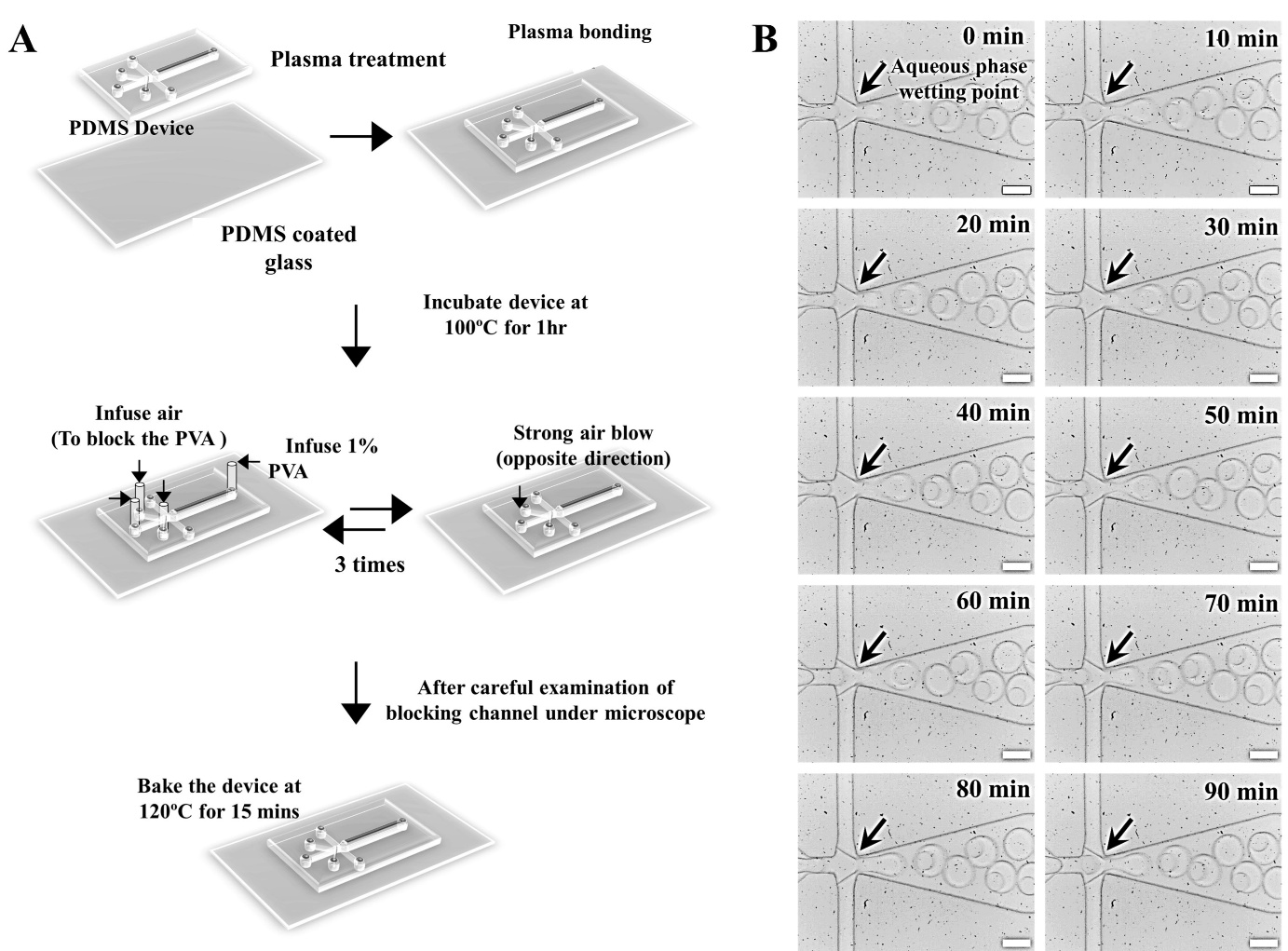


**Figure S2.** Region-selective modification of the PDMS surface for the generation of DE droplets. (A) Schematic representation of the PDMS surface treatment process using 1% PVA inside the microchannel. (B) Optical images showing the stable production of DE droplets over 90 min in the selectively modified PDMS microfluidic device. Flow rates were set as follows: Q_W1_ = 100 µl/hr (inner aqueous phase), Qo = 200 µl/hr (middle oil phase), and Q_W2_ = 400 µl/hr (outer aqueous phase). Here, W1 represents the inner aqueous phase, O represents the middle oil phase, and W2 represents the outer aqueous phase. All scale bars represent 100 µm. DE, double emilsion; PDMS, polydimethylsiloxane; PVA, polyvinyl alcohol


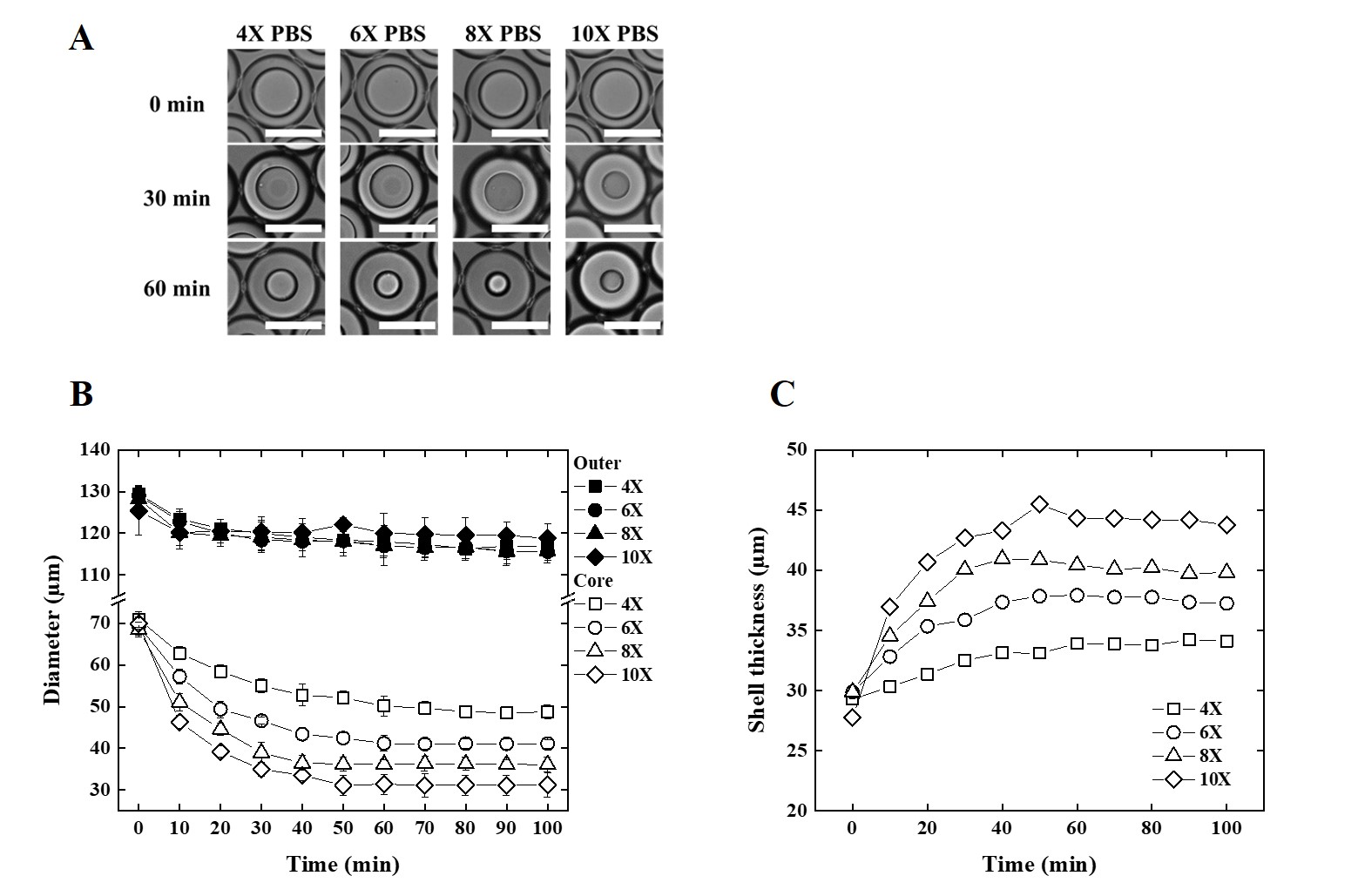
**Figure S3.** The change in outer and core diameter of DE droplets. (A) Time-lapse optical images of the shrinkage of DE droplets under different strengths of the outer buffer (4×, 6×, 8×, and 10× PBS). (B) Quantitative measurement of changes in the outer and core diameters as a function of time under various outer buffers. (C) The change in shell thickness as a function of time. All scale bars indicate 100 µm. DE, double emulsion; PBS, phosphate-buffered saline


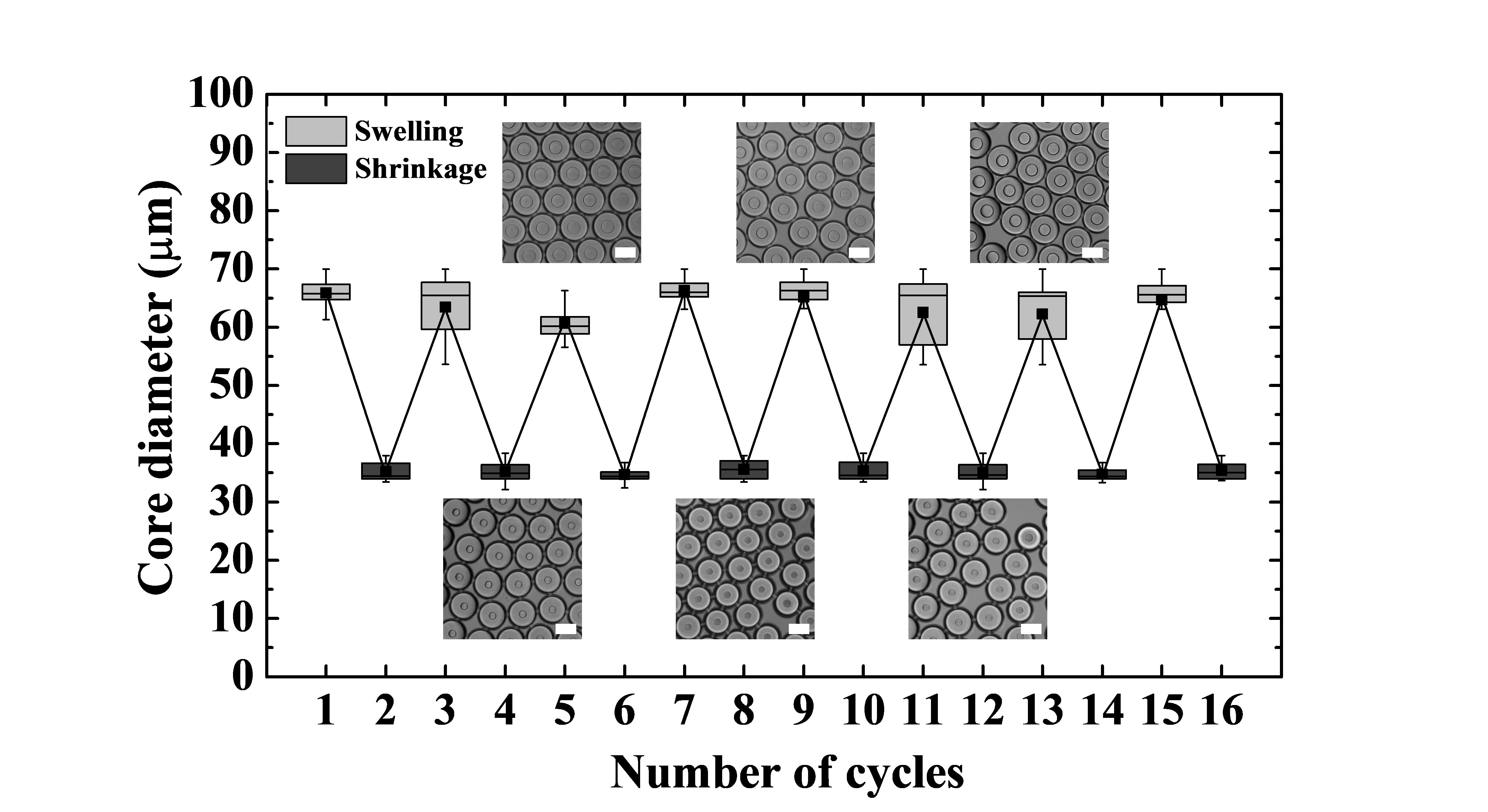


**Figure S4.** Reversible actuation of DE droplets. Optical images of DE droplets between shrinkage and swelling. In this experiment, DE droplets were exposed to 8× PBS buffer to induce shrinkage and subsequently transferred to 1×PBS buffer to promote swelling. All scale bars represent 100 µm. All error bars represent standard deviation. DE, double emulsion; PBS, phosphate-buffered saline


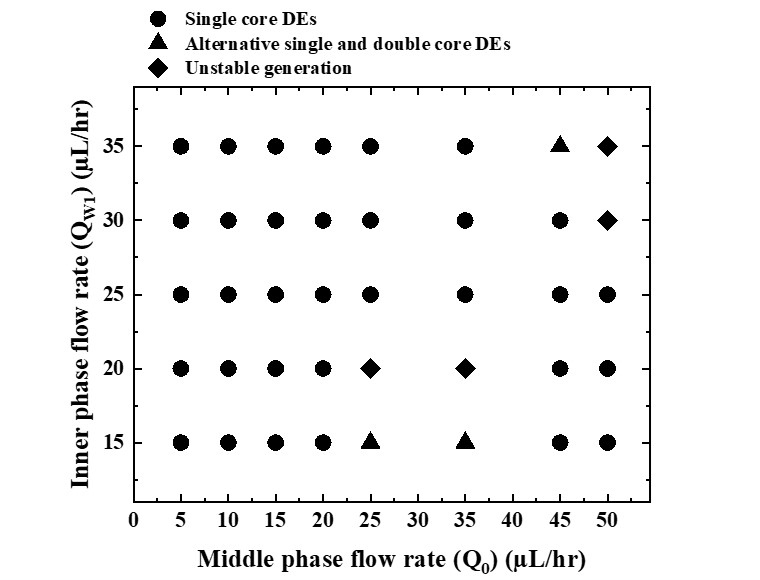


**Figure S5**. Phase diagram illustrating the generation of DE droplets across varying inner (Q_W1_) and middle (Q_o_) phase flow rates, while maintaining a constant outer phase (Q_W2_) flow rate of 200 µl/hr. Solid circular markers represent conditions that produce monodisperse single-core DE droplets, whereas solid non-circular markers denote unstable conditions. At the optimum flow rate, we obtained the dripping regime in which the formation of DE droplets is dominated by two forces that are the shear force that drags the drop and capillary force that holds the drop in the tip, which results in the production of thin-shell DE droplets. DE, double emulsion





**Figure S6.** Representative fluorescent images of DE droplets encapsulating the EPO gene (600 copies/µl) in blood samples spiked from three different individuals. All scale bars represent 100 µm. DE, double emulsion


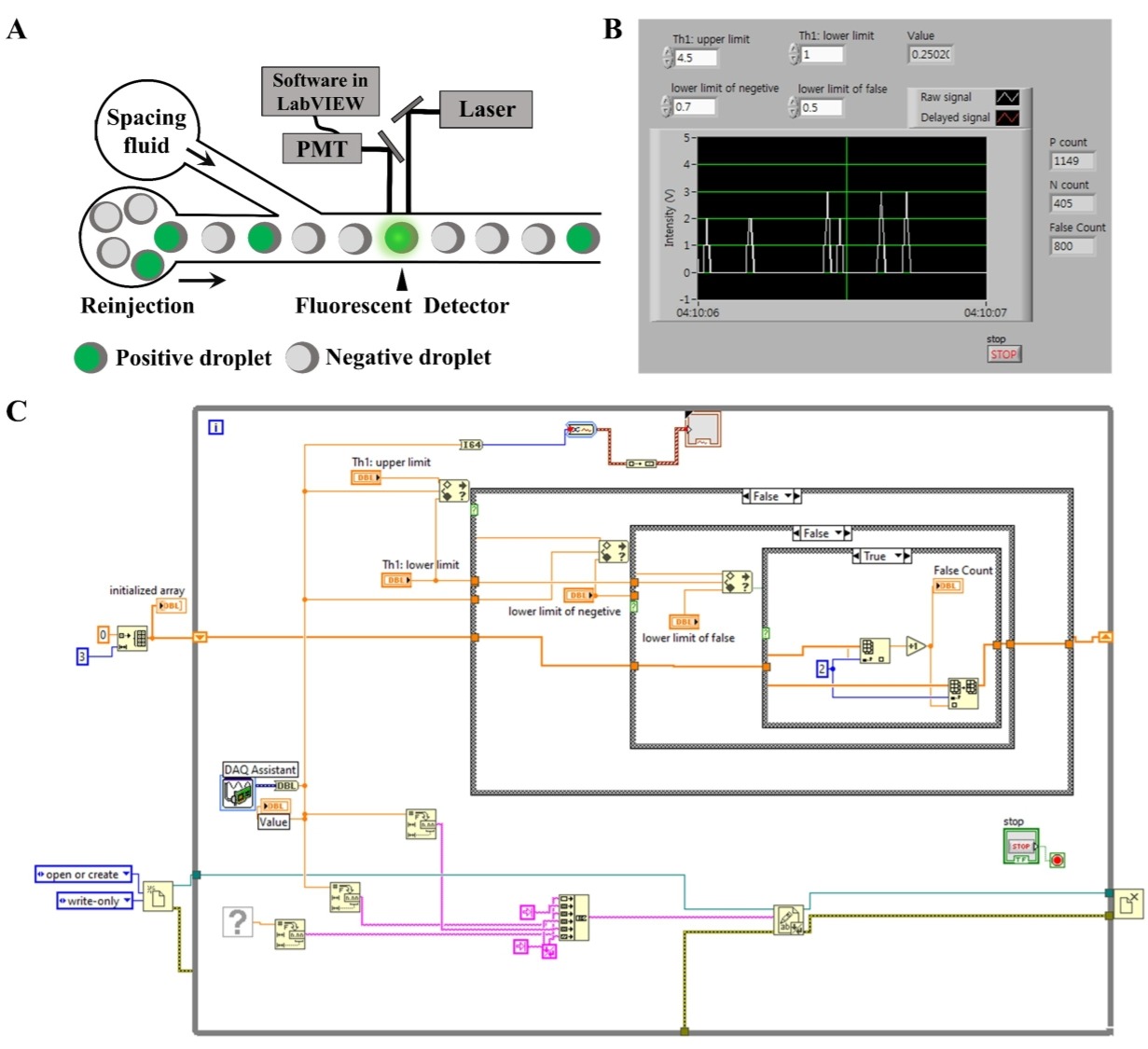


**Figure S7.** LabVIEW program for data acquisition. (A) Schematic diagram showing the process of on-chip screening of positive and negative droplets. The laser emitted light at 488 nm, directed through a multi-edge dichroic beam splitter to the microscope. Inside the microscope, the laser light passes through a beam splitter and is reflected up into the objective by a conventional mirror. The fluorescent emission from each droplet passed back along the path of the laser beam. It is then reflected by the dichroic beam splitter to the sensor of the PMT via a bandpass filter. The signal output from the PMT was analyzed using a program written in LabView 8.2 which allowed the identification of droplets by fluorescence peaks. (B) User interface and data visualization. This interface allows users to adjust critical parameters, such as the upper and lower thresholds for data processing. Real-time modifications can be made to adapt to varying experimental conditions or data quality requirements. The graphical display shows a time course of signal intensities, providing a visual representation of data as it is being processed. Displays count for various categories, such as "P count" (positive counts), "N count" (negative counts), and "False Count" (erroneous detections), offering a quick statistical overview of the signal classification performance. (C) Logic diagram of signal processing and threshold management for the measurement of positive and negative DE droplets. DE, double emulsion; PMT, photomultiplier tube


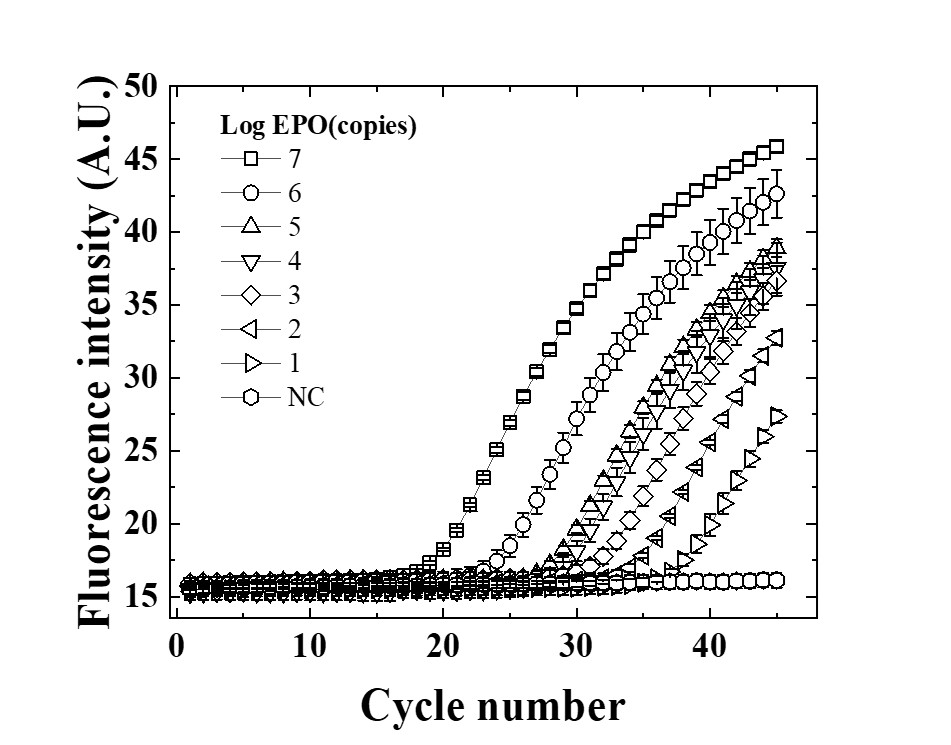


**Figure. S8**. qPCR detection of the *hEPO* gene (n = 3), showing a dynamic range from 10⁷ to 10 copies in pure water. NC: negative control, blank sample.

**Table S1.** Comparison of qPCR, ddPCR, and amplification-free CRISPR-based methods with the osmotically tunable droplet platform.

| **Method** | **Sample** | **Limit of Detection**  **(LOD)** | **Assay Time** | **Nucleic Acid Extraction** | **Amplification Required** | **References** |
| --- | --- | --- | --- | --- | --- | --- |
| qPCR | Extracted  nucleic acids | ~1,500 copies in biological matrices | ~3h  (plus extraction) | Required | Yes | [S1]  Figure S8 |
| ddPCR | Extracted DNA/RNA from blood/urine | ~10 – 50 copies | ~3 – 4 h (plus extraction) | Required | Yes | [S2] |
| Amplification-free CRISPR (DETECTR) | Extracted  nucleic acids | ~1000 – 2000 copies | 1 – 2 h | Sometimes required | No | [S3] |
| Amplification-free CRISPR (SHERLOCK) | Extracted  nucleic acids | ~500 – 1000 copies | 1 – 2 h | Sometimes required | No | [S4] |
| Osmotically tunable DE droplet platform (This work) | Direct serum | 600 copies | ≤ 1 h | Not required | No | This work |

DE, double emulsion

**References**

[S1] K. S. Wong, H. W. Cheung, C. W. Szeto, et al., "A multiplex qPCR assay for transgenes detection: A novel approach for gene doping control in horseracing using conventional laboratory setup*"* *Drug Testing and Analysis (***2023)**, *15* (8), 879, <https://doi.org/10.1002/dta.3483>

[S2] D. A. Moser, L. Braga, A. Raso, et al., "Transgene detection by digital droplet PCR*"* *PLoS One (***2014)**, *9* (11), e111781, <https://doi.org/10.1371/journal.pone.0111781>

[S3] J. S. Chen, E. Ma, L. B. Harrington, et al., "CRISPR-Cas12a target binding unleashes indiscriminate single-stranded DNase activity*"* *Science (***2018)**, *360* (6387), 436, [10.1126/science.aar6245](https://doi.org/10.1126/science.aar6245).

[S4] J. S. Gootenberg, O. O. Abudayyeh, M. J. Kellner, et al., "Multiplexed and portable nucleic acid detection platform with Cas13, Cas12a, and Csm6*"* *Science (***2018)**, *360* (6387), 439, [10.1126/science.aaq0179](https://doi.org/10.1126/science.aaq0179).
